# Supplementary material for: Nature-Based Early Childhood Education and Children’s Social, Emotional and Cognitive Development: A Mixed-Methods Systematic Review
Source: Int J Environ Res Public Health. 2022 May 13;19(10):5967. doi: 10.3390/ijerph19105967 (PMC9142068; doi:10.3390/ijerph19105967)
Supplement: Supplementary file 1 [file ijerph-19-05967-s001.zip › ijerph-1691946-supplementary.pdf]

Additional files

Additional file S1. Example search strategy for the Medline database.

S1	(MH "Child, Preschool")
S2	TI child* OR AB child*
S3	TI (boy* OR girl*) or AB (boy* OR girl*)
S4	TI toddler OR AB toddler
S5	TI young N1 child* OR AB young N1 child*
S6	TI early N1 child* OR AB early N1 child*
S7	TI early N1 year* OR AB early N1 year*
S8	TI “pre-primary” or AB “pre-primary”
S9	S1 OR S2 OR S3 OR S4 OR S5 OR S6 OR S7 OR S8
S10	(MH "Schools, Nursery")
S11	TI nurser* OR AB nurser*
S12	(MH "Learning") OR TI early N1 learning OR AB early N1 learning
S13	TI (“preschool” or “pre-school”) OR AB (“preschool” or “pre-school”)
S14	TI kindergarten OR AB kindergarten
S15	TI (childcare OR child N1 care) OR AB (childcare OR child N1 care)
S16	TI (daycare OR day N1 care) OR AB (daycare OR day N1 care)
S17	(MH "Education") OR TI (education OR "preschool education" OR "outdoor education" OR "adventure education") OR AB (education OR "preschool education" OR "outdoor education" OR "adventure education")
S18	MM "Play and Playthings" OR TI (Play OR “play-based learning”) OR AB (Play OR “play-based learning”)
S19	TX (Waldkindergartens OR udeskole OR friluftsliv OR peuterspeelzaal OR kinderopvang OR bush N1 kinder*) OR TI (forest N1 kindergarten* OR forest N1 school*) OR AB (forest N1 kindergarten* OR forest N1 school*)
S20	S10 OR S11 OR S12 OR S13 OR S14 OR S15 OR S16 OR S17 OR S18 OR S19
S21	TI outdoor* OR AB outdoor*
S22	TI (nature OR “nature-based”) OR AB (“nature-based”)
S23	TI environment* OR TI outdoor N1 environment* OR AB outdoor N1 environment*
S24	TI (forest* OR wood* OR park* OR recreation* OR landscape* OR tree* OR hill* OR garden* OR beach* OR eco)
S25	AB (forest* OR wood* OR park* OR recreation* OR landscape* OR tree* OR hill* OR garden* OR beach* OR eco)
S26	TI (green OR greenspace or green N1 space) OR AB (green OR greenspace or green N1 space)
S27	TI (loose N1 parts OR “loose-parts”) OR AB (loose N1 parts OR “loose-parts”)
S28	TI (adventure* OR wild OR “open-air”) OR AB (adventure* OR wild OR “open-air”)
S29	S21 OR S22 OR S23 OR S24 OR S25 OR S26 OR S27 OR S28
S30	S9 AND S21 AND S30

Additional file S2. Quality appraisal tools

Modified Effective Public Health Practice Project (EPHPP) Quality Assessment Tool

A) SELECTION BIAS

(Q1) Are the individuals selected to participate in the study likely to be representative of the target population?

(i.e. children aged 2-7 years not in formal education yet)

1. Very likely
2. Somewhat likely
3. Not likely
4. Can't tell

(Q2) What percentage of selected individuals consented to the research?

1. 80 - 100% agreement
2. 60 – 79% agreement
3. less than 60% agreement
4. Not applicable
5. Can't tell

RATE THIS SECTION	STRONG	MODERATE	WEAK
See dictionary	1	2	3

B) STUDY DESIGN

Indicate the study design:

1. Randomized controlled trial
2. Controlled clinical trial
3. Cohort analytic (two group pre + post)
4. Case-control
5. Cohort (one group pre + post (before and after))
6. Interrupted time series
7. Other specify ____________________________
8. Can't tell

Was the study described as randomized? If NO, go to Component C.

No **Yes**

If Yes, was the method of randomization described? (See dictionary)

No **Yes**

If Yes, was the method appropriate? (See dictionary)

No **Yes**

RATE THIS SECTION	STRONG	MODERATE	WEAK
See dictionary	1	2	3

C) CONFOUNDERS

(Q1) Were there important differences between groups prior to the intervention?

1. Yes

2. No
3. Can’t tell

The following are examples of confounders:

1. Gender
2. Age
3. Socio economic status (SES – e.g. Parental education, deprivation status)

(Q2) If yes, indicate the percentage of relevant confounders that were controlled (either in the design (e.g. stratification, matching) or analysis)?

1. All confounders
2. Two confounders
3. One confounder
4. Can’t Tell

RATE THIS SECTION	STRONG	MODERATE	WEAK
See dictionary	1	2	3

D) BLINDING

(Q1) Was (were) the outcome assessor(s) and/or analysists aware of the intervention or exposure status of participants?

1. Yes
2. No
3. Can’t tell

(Q2) Were outcome assessors aware of the research question?

1. Yes
2. No
3. Can’t tell

RATE THIS SECTION	STRONG	MODERATE	WEAK
See dictionary	1	2	3

E) DATA COLLECTION METHODS

(Q1) Were data collection tools shown to be valid?

1. Yes
2. No
3. Can’t tell

(Q2) Were data collection tools shown to be reliable?

1. Yes
2. No
3. Can’t tell

RATE THIS SECTION	STRONG	MODERATE	WEAK
See dictionary	1	2	3

F) WITHDRAWALS AND DROP-OUTS

(Q1) Were withdrawals and drop-outs reported in terms of numbers and/or reasons per group?

1. Yes
2. No
3. Can't tell
4. Not Applicable (i.e. one time surveys or interviews)

(Q2) Indicate the percentage of participants completing the study. (If the percentage differs by groups, record the lowest).

1. 80 -100%
2. 60 - 79%
3. less than 60%
4. Can't tell
5. Not Applicable (i.e. Retrospective case-control)

RATE THIS SECTION	STRONG	MODERATE	WEAK
See dictionary	1	2	3

COMPONENT RATINGS

Please transcribe the information from the grey boxes on pages 1-3 onto this page. See dictionary on how to rate this section.

**A**	**SELECTION BIAS**	**STRONG**	**MODERATE**	**WEAK**
		1	2	3
**B**	**STUDY DESIGN**	**STRONG**	**MODERATE**	**WEAK**
		1	2	3
**C**	**CONFOUNDERS**	**STRONG**	**MODERATE**	**WEAK**
		1	2	3
**D**	**BLINDING**	**STRONG**	**MODERATE**	**WEAK**
		1	2	3
**E**	**DATA COLLECTION METHOD**	**STRONG**	**MODERATE**	**WEAK**
		1	2	3
**F**	**WITHDRAWALS AND DROPOUTS**	**STRONG**	**MODERATE**	**WEAK**
		1	2	3

Overall Grade (based on above six criteria):

• Scored **1** for study design (i.e. controlled studies); AND<br>• Scored **1** or **2** in at least <u>three</u> other important components, including:<br>o selection bias<br>o confounders<br>o blinding<br>o withdrawals and drop-outs.	**STRONG**<br>**1**
• Scored **1** for study design; AND<br>• Scored **1** or **2** in <u>two</u> other important components, including:<br>o selection bias<br>o confounders<br>o blinding<br>o withdrawals and drop-outs.<br><br>OR<br><br>• Scored **2** for study design; AND<br>• Scored **1** or **2** in at least <u>three</u> other important components, including:<br>o selection bias<br>o confounders<br>o blinding<br>o withdrawals and drop-outs.	**MODERATE**<br>**2**
• Scored **1** for study design; AND<br>• Scored **3** in more than <u>two</u> other important components, including:<br>o selection bias<br>o confounders<br>o blinding<br>o withdrawals and drop-outs.<br><br>OR<br><br>• Scored **2** for study design; AND<br>• Scored **3** in more than <u>one</u> other important components, including:<br>o selection bias<br>o confounders<br>o blinding<br>o withdrawals and drop-outs.<br><br>OR<br>• Scored **3** for study design	**WEAK**<br>**3**

Dixon-Woods (2004) checklist

**Question 1**	Are the research questions clear?
**Question 2**	Are the research questions suited to qualitative inquiry
**Question 3**	Are the following clearly described?<br>- Sampling<br>- Data collection<br>- Analysis
**Question 4**	Are the following appropriate to the research question?<br>- Sampling

	- Data collection<br>- Analysis
**Question 5**	Are the claims made supported by sufficient evidence?
**Question 6**	Are the data, interpretations, and conclusions clearly integrated?
**Question 7**	Does the paper make a useful contribution to the review question?

Additional file S3. Quality of included quantitative studies as assessed by the EPHPP tool

Study ID	Selection bias	Study design	Confounders	Blinding	Data collection methods	Withdrawals and drop-outs	Final Grade
Agostini et al (2018) [59]	3 = Weak	1 = Strong	3 = Weak	3 = Weak	1 = Strong	3 = Weak	**3 = Weak**
Barrable et al (2020) [57]	3 = Weak	3 = Weak	3 = Weak	3 = Weak	1 = Strong	N/A	**3 = Weak**
Brussoni et al (2017) [48]	2 = Moderate	2 = Moderate	2 = Moderate	3 = Weak	1 = Strong	1 = Strong	**2 = Moderate**
Carrus (2012) [60]	3 = Weak	3 = Weak	3 = Weak	3 = Weak	3 = Weak	N/A	**3 = Weak**
Cloward Drown & Christensen (2014) [36]	3 = Weak	3 = Weak	1 = Strong	3 = Weak	1 = Strong	N/A	**3 = Weak**
Cooper (2018) [56]	3 = Weak	1 = Strong	3 = Weak	3 = Weak	1 = Strong	3 = Weak	**3 = Weak**
Cordiano et al (2019) [26]	3 = Weak	1 = Strong	3 = Weak	3 = Weak	1 = Strong	3 = Weak	**3 = Weak**
Cosco et al (2014) [35]	1 = Strong	2 = Moderate	1 = Strong	3 = Weak	1 = Strong	3 = Weak	**3 = Weak**
Dyment et al (2013) [42]	1 = Strong	3 = Weak	3 = Weak	3 = Weak	1 = Strong	N/A	**3 = Weak**
Elliot et al (2014) [46]	3 = Weak	1 = Strong	2 = Moderate	3 = Weak	1 = Strong	1 = Strong	**2 = Moderate**
Ernst (2014) [28]	2 = Moderate	3 = Weak	3 = Weak	3 = Weak	3 = Weak	N/A	**3 = Weak**
Ernst & Burcak (2019) [30]	3 = Weak	1 = Strong	2 = Moderate	3 = Weak	1 = Strong	3 = Weak	**3 = Weak**
Ernst et al (2019) & Ernst & Burcak (2019) [29, 30]	3 = Weak	2 = Moderate	2 = Moderate	3 = Weak	1 = Strong	3 = Weak	**3 = Weak**
Wojciehowski & Ernst (2018) & Ernst & Burcak (2019) [30, 31]	3 = Weak	2 = Moderate	2 = Moderate	3 = Weak	1 = Strong	3 = Weak	**3 = Weak**
Burgess & Ernst (2020) [27]	3 = Weak	1 = Strong	2 = Moderate	3 = Weak	1 = Strong	3 = Weak	**3 = Weak**
Zamzow & Ernst (2020) & Ernst & Burcak (2019) [30, 32]	3 = Weak	1 = Strong	2 = Moderate	3 = Weak	1 = Strong	3 = Weak	**3 = Weak**
Fyfe-Johnson et al (2019) [33]	3 = Weak	3 = Weak	3 = Weak	3 = Weak	1 = Strong	N/A	**3 = Weak**
Giusti et al (2014) [54]	2 = Moderate	3 = Weak	1 = Strong	3 = Weak	3 = Weak	N/A	**3 = Weak**
Lillard (2016) [38]	3 = Weak	2 = Moderate	1 = Strong	3 = Weak	1 = Strong	1 = Strong	**3 = Weak**
Luchs, & Fikus (2013) [62]	3 = Weak	3 = Weak	3 = Weak	3 = Weak	3 = Weak	N/A	**3 = Weak**
Mårtensson et al (2009) [53]	3 = Weak	3 = Weak	1 = Strong	3 = Weak	1 = Strong	N/A	**3 = Weak**
Morrissey et al (2017) [43]	3 = Weak	3 = Weak	3 = Weak	3 = Weak	3 = Weak	N/A	**3 = Weak**
Müller et al (2017) [47]	3 = Weak	1 = Strong	3 = Weak	3 = Weak	1 = Strong	1 = Strong	**3 = Weak**
Nazaruk & Klim-Klimaszewska (2017) [63]	3 = Weak	2 = Moderate	3 = Weak	3 = Weak	3 = Weak	1 = Strong	**3 = Weak**

Park et al (2016) [64]	3 = Weak	2 = Moderate	3 = Weak	3 = Weak	1 = Strong	1 = Strong	**3 = Weak**
Rice & Torquati (2013) [34]	3 = Weak	3 = Weak	3 = Weak	3 = Weak	1 = Strong	3 = Weak	**3 = Weak**
Robertson et al (2020) [41]	3 = Weak	3 = Weak	3 = Weak	3 = Weak	3 = Weak	N/A	**3 = Weak**
Sando (2019) [50]	2 = Moderate	3 = Weak	2 = Moderate	3 = Weak	1 = Strong	3 = Weak	**3 = Weak**
Söderström at al (2013) [55]	2 = Moderate	3 = Weak	1 = Strong	3 = Weak	1 = Strong	N/A	**3 = Weak**
Yılmaz et al (2020) [65]	3 = Weak	2 = Moderate	3 = Weak	3 = Weak	2 = Moderate	1 = Strong	**3 = Weak**
Zamani (2013) [37]	3 = Weak	3 = Weak	1 = Strong	3 = Weak	2 = Moderate	N/A	**3 = Weak**

Additional file S4. Characteristics of included studies

Quantitative

Table S1. Characteristics of included quantitative studies

**Author, year and country**	**Study design**	**Age (range or mean ± SD), sex (n or % m/f), SES.**	**Exposure and comparison**	**Follow-up time point**	**Outcome(s)**	**Data analysis**
**Nature-based ECE**						
Agostini et al (2018), Italy [59].<br><br>E: 41 children / 7 teachers / 1 school<br><br>C: 52 children / 13 teachers / 1 school	Controlled before & after	E:<br>Age: 47.2 months ± 6.52<br>Gender: 13m/28f<br><br>C:<br>Age: 46.75 months ± 6.95<br>Gender: 29m/23f<br><br>SES not reported.	E: Teachers underwent special training in outdoor education over one year including (15 days). ECE consisted of a green park with some centuries-old trees (e.g., firs, willows, maples), plants and flowers, and without any play structures.<br><br>C: ECE contained grass and cement without larger plants, trees, and play structures.	T1= Jan 2014<br>T2= May 2014<br>T3= Oct 2014<br>T4= May 2015 (16 months from baselines)	Cognitive<br>Social and Emotional<br>Nature Connectedness<br>Play	Mixed-Model Repeated Measures analysis of variance (ANOVA)
Cooper (2018), United Kingdom (England) [56].	Controlled before & after	E:<br>Age: 47 months (range 45-48)	E: Forest school sessions run by two trained leaders which operate for 10 week cycles on Tuesday AM and PM (2 hours each). Children attend either the AM or	10- weeks	Cognitive<br>Social and Emotional	Wilcoxon Signed-Rank Test; Mann-

E: 13 children<br><br>C: 11 children<br><br>Children from the same school		Gender: 7m/4f<br><br>C:<br>Age: 44 months (range 41-47)<br><br>Gender: 7m/4f<br><br>SES was noted as being “generally above average" for both groups.	PM session. The forest school consists of trees and vegetation, a seating area made from logs, a mud kitchen using old crates and a tyre, a greenhouse and pond. The forest school is located on site and when children do not have forest school sessions outdoors, they have a “ free flow” environment where children are allowed outside when they want.<br><br>C: Usual nursery practice which also involves a large amount of outdoor exploration. Children also participated in a one hour per week nature play session which incorporated elements of the forest school and included gardening, litter picking and PA. Staff have created an engaging multi-sensory outdoor environment for children which includes a sand pit area, water features and climbing apparatus. The nursery has an allotment system for children to plant fruit trees.			Whitney U test.
Cordiano et al (2019), USA [26].<br><br>E: 12 children / 1 ECE class.<br><br>C: 14 children / 1 class.<br><br>Children from the same school.	Controlled before & after study	Age: 51.5 months (4.3 years)<br><br>Gender: 26f<br><br>SES: 46% of students attending the ECE receive financial assistance	E: Outdoor pre-primary programme involved children spending five mornings per week at the school’s outdoor campus. The children were outdoors in the forest for 90% of the school day.<br><br>C: Traditional prekindergarten programme involved children spending five mornings per week at the school’s main campus. This involves an Eco!Wonder curriculum that teaches all children about nature and sustainability. Children also visited the outdoor campus one morning per week and spent one immersion week at the outdoor campus in the spring. The remainder of	8 months	Cognitive<br>Social and Emotional<br>Play	Mixed-model analysis of covariance (ANCOVA)<br><br>Covariates: age

			their outdoor time was spent in built environments.			
Elliot et al (2014), Canada [46].<br><br>E: 21 children / 1 ECE<br><br>C: 22 children / 2 ECE	Controlled before & after (mixed-methods)	E:<br>Age: 5.3 years (0.5 SD)<br><br>Gender: 10m/11f<br><br>SES not reported.<br><br>C:<br>Age: 5.3 years (0.3 SD)<br><br>Sex: 7m/15f<br><br>SES not reported.	E: A two-year pilot project in which 22 students would spend the mornings from 9:00 to 11:45 outside their school, exploring their local natural environment.<br><br>C: not described	6 months	Nature Connectedness	ANOVA
Ernst & Burcak (2019), USA [30].<br><br>E: 34 children / 2 ECE C: 43 children / 2 ECE<br><br>Burgess & Ernst (2020), USA [27].<br><br>E: 84 children / 4 ECE<br>C: 24 children / 2 ECE<br><br>Zamzow & Ernst (2020) [32].<br><br>E: 78 / 4 ECE<br>C: 44 children / 2 ECE	Controlled before & after	E:<br>Age: 4 years<br><br>Sex: 50%m/ 50%f<br><br>C<br>Age: 4 years<br><br>Sex:64%m/ 36%f<br><br>SES not reported	E: The nature-preschools utilised a combination of wild natural settings spaces that were minimally managed and natural playscapes designed specifically for nature play. The majority of time spent was in free play outdoors in unmaintained or minimally maintained natural settings regardless of weather conditions (approximately four to five hours per day).<br><br>C: Non-nature preschools emphasised child-directed play. The majority of time was spent indoors in free or loosely guided play (four to five hours), with about one hour daily of teacher-led playful learning.<br><br>Children at both groups had one to two hours of daily outdoor playtime (weather permitting) in a maintained outdoor space that contained playground equipment.	9 months	Cognitive (all 5 papers)<br><br>Social and emotional (Ernst & Burcak, 2019l Ernst et al., 2019)<br><br>Play (Burgess & Ernst, 2020)	GLM<br><br>Covariates: pre-test scores, age, gender, prior participation<br><br>t-test

Ernst et al (2019) [29].<br><br>E: 78 children / 4 ECE<br><br>Wojciehowski & Ernst (2018) [31] .<br>E: 75 children / 4 ECE	Uncontrolled before & after					
Müller et al (2017), Canada [47].<br><br>E: 43 children / 1 ECE<br><br>C: 45 children / 1 ECE	Controlled before & after	Age:<br><br>E: 63.56 months (3.33 SD)<br>C: 64 months (3.56 SD)<br><br>Gender not reported.<br><br>SES not reported.	E: "nature kindergarten"<br><br>C: "traditional kindergarten"<br><br>Neither are described.	9 months Sep/Oct-May	Cognitive<br>Social and Emotional<br>Nature Connectedness	Analyses of Covariance (ANCOVA)
Nazaruk & Klim-Klimaszewska (2017), Poland [63].<br><br>E: 90 children (50 urban / 40 rural)	Uncontrolled before & after	Age: 6 years<br><br>Gender not reported.<br><br>SES not reported.	Teachers arranged trips in the forest, the park, the allotment garden, the meadow, the agritourism farm, animals at the zoo.	6 months	Nature connectedness	Mann-Whitney U test; Pearson Chi test
Yilmaz et al (2020), Turkey [65].<br><br>40 children / 1 ECE	Uncontrolled before & after	Age: 72 months (6 years)<br><br>Gender: 14m/26<br><br>SES not reported.	E: Children visited a natural, unstructured area for one day in a week for four consecutive weeks.<br><br>The education programme consisted of 12 semi-structured activities (3 per week).<br><br>In addition, children also had 30 minutes walk near a natural pond when they visit	4 weeks (1 session per week - 1 full day) conducted in spring 2018	Nature connectedness	Paired sample t-test; ANOVA

			the setting each week and each week, children had 30 minutes unstructured free play time to discover the natural environment.			
Barrable et al (2020), UK (England, Scotland, Wales) [57].<br><br>E: 141 /12 ECE<br><br>C: 110 children / 6 ECE	Controlled cross-sectional	Age: 4.53 ± 1.39<br><br>Gender: 127m/89f<br><br>SES not reported.	E: ECE's that have a continuous outdoor provision, with no permanent indoor access and children are outdoors for the whole duration of the ECE day.<br><br>C: ECE's that are predominately indoor and have variable outdoor provision.	N/A	Nature connectedness	GLM with a binomial error distribution<br><br>Covariates: Parental NC scores, sex, exposure
Fyfe-Johnson et al (2019), USA [33].<br><br>E: 20 children / 1 ECE<br><br>C: 13 children (waitlist control or 2-hour nature-based, outdoor enrichment class provided by experimental ECE	Controlled cross-sectional	Age: 3-5 years<br><br>Gender:<br>E: 11m/9f<br>C: 9m/4f<br><br>SES:<br>E: 18 > $90,000<br>C: 8> $90,000	E: The nature ECE occurs outdoors in a forested park where most children attend 5 days per week from 9 am to 1 pm; 2-day and 3-day per week options are available on a limited basis. The physical environment consists of dedicated classroom areas in the forested areas. Children use logs and tree stumps to sit; portable canopies are used during inclement weather. Most of the day is spent hiking and exploring the surrounding forest. No traditional play structures or pre-fabricated playgrounds are utilized.<br>C: 2 hour nature-based outdoor enrichment class was offered once weekly by the same nature ECE the intervention group children attended. Classes were led by a teacher and attended by both child and caregiver. The classes consisted of science-based exploration through outdoor play in a forested park and involved: circle time, station time (learning stations that emphasize sensory and fine motor skills, creativity, and numerical and literacy	N/A	Cognitive<br>Social and emotional	Descriptives only.

			skills), short stories, and hikes. Others were included in a wait-list control			
Giusti et al (2014), Sweden [54].<br><br>E: 11 children / 2 ECE<br><br>C: 16 children / 5 ECE	Controlled cross-sectional	Age: 5 years<br><br>Gender not described.<br><br>SES not reported.	ECE were assessed on their frequency of natural experiences. Each ECE was ranked according to the highest frequency of use of the greatest variety of nature experiences in its surroundings<br><br>E: The ten ECE with the most frequent use of all nature experiences.<br><br>C: The ten ECE with the least frequent use of all nature experiences.	N/A	Nature connectedness	t-test
Rice & Torquati (2013), USA [34].<br><br>E: 68 children / 6 ECE<br><br>C: 46 children /4 ECE	Controlled cross-sectional	Age: 56.4 months (12.8 SD)<br><br>Gender not reported.<br><br>SES: 46.5% of participants reported an annual income of $85,000 or more.	E: The nature programme featured: vegetation, gardens, areas for digging in soil, sand, and “loose parts” (sticks, seeds, pinecones etc) and other naturally occurring objects that children used in their play. Climbing structures and pretend play structures such as a boat or a playhouse were also included.<br><br>C: The non-nature programmes consisted of pretend play structures, sand and/or wood chips, and paved surfaces for wheeled toys, and had few natural elements such as trees or grass.	N/A	Nature connectedness	ANOVA and Chi square
Robertson et al (2020), Australia [41].<br><br>E: 15 children / 1 ECE<br><br>C: 15 children / 1 ECE	Controlled cross-sectional	Age: 4-5 years<br><br>Gender not reported.<br><br>SES not reported.	E: ECE is in a rural area and consisted of a small traditional playground area (sand pit, obstacle course etc.) and a larger open ended nature area consisting of trees, shrubbery, grass, natural loose-parts). It has a highly naturalised area towards the rear that was rich in natural elements including small and large shrubbery, and larger tree and vegetation	N/A	Play	Independent samples t-test

			C: ECE is located in a suburban area and consisted predominately of man-made structures (almost half the space). The playground also consisted of some nature such as trees and vegetable garden.			
Ernst (2014), USA [28].<br><br>E: 46 educators	Cross-sectional	Not described.	Outdoor environments that range from relatively natural to wild spaces.	N/A	Cognitive<br>Social and emotional<br>Nature connectedness	Multiple regression
**ECE natural playgrounds**						
Brussoni et al (2017), Canada [48].<br><br>E: 48 children / 2 ECE	Uncontrolled before & after (mixed methods)	Age: 4.28 (0.63 SD)<br><br>Gender: 53% m/47%f<br><br>SES not reported.	Playgrounds were improved using the Seven Cs which consists of 27 items, rated on a 5-point scale, for a maximum score of 135<br><br>Changes predominately involved inclusion of more natural elements such as, vegetation, boulders, rock, loose parts. Seven Cs scores increased from 44 to 97 in ECE A, and 35 to 125 in ECE B.	Data were collected at T2; May-July 2014) two-weeks after playground modification	Social and emotional<br>Play	Wilcoxon signed rank tests; General linear modelling.<br><br>Covariates: age, gender, ECE
Cosco et al (2014), USA [35].<br><br>E: 804 / 27 ECE	Uncontrolled before & after	Age: 2-5 years<br><br>Gender not reported.<br><br>SES not reported.	Preventing Obesity by Design is an ECE outdoor renovation intervention. Prior to the intervention the space had few structures (slides, swings etc.) in a rectangle space enclosed by a fence. Whereas, post intervention, the space had more natural elements, including trees, garden, vegetation etc.	Not described.	Social and emotional	Logistic regression and bivariate correlations<br><br>Covariates: gender
Cloward Drown et al (2014), USA [36].<br><br>E: 24 children / 1 ECE<br>(observed in 2 different playgrounds,	Controlled cross-sectional	Age: 4.5 years<br><br>Gender: 7m/17f<br><br>SES not reported.	E: The natural playground was characterised by a majority of natural surfaces (vegetation, boulders, grass etc.) This playground also consists of sandbox, bikes pathway and instruments.<br><br>C: The manufactured playground is equipment-oriented with hard surfaces. Although it includes some vegetation, the	N/A	Play	Chi-squared

natural vs manufactured)			main features are a xylophone, slide, and pit, a ball pit, water play area and concrete ramps leading to a plastic play castle and a spin chair.			
Luchs, & Fikus (2013), Germany [62].<br><br>E: 38 children / 1 ECE<br><br>C: 21 children / 1 ECE	Controlled cross-sectional	Age: 5-6 years<br><br>Gender: 33m/26f<br><br>SES not described	E: the natural playground provides children with wild and natural areas, including trees, grass, flowers etc. There are also sandboxes, dirt, rock and water and mud area.<br><br>C: the contemporary playground provides traditional man-made structures, such as slide, sandbox, playhouse, water area, seesaw, roundabout etc.	N/A	Play	t-test
Carrus (2012), Italy [60].<br><br>E: 16 children / 1 ECE	Cross-sectional	Age: 18-36 months (1.5-3 years). Gender not reported.<br><br>SES not reported.	Free play in garden and green spaces of the ECE compared to free play indoors.	N/A	Cognitive<br>Social and emotional	mixed model ANOVA with 2-way interactions
Dyment et al (2013), Australia [42].<br><br>E: 120 children / 3 ECE<br><br>C: 40 children / 1 ECE	Cross-sectional	Age: ECE A = older toddlers, young children; ECE B = young children; ECE C = older toddlers, young children, ECE D = 2-5 year olds<br><br>Sex: 57%m/ 43% f.<br><br>SES: the 4 centres differed in terms of SES (Centre A = high SES, B= varied SES, C= low SES, D= medium)	E: three centres all of which contained natural areas (trees, rocks, gardens). Two ECE's also has manufactured elements<br><br>C: one centre which contained no natural areas	N/A	Play	Descriptives only.
Morrissey et al (2017), Australia [43].	Cross-sectional	Age: 4-5 years<br><br>Gender: 28m/28f	E: ECE contained natural structures such as logs, shrubs, rocks etc. It also contains a few manmade elements.	N/A	Play	Chi-square analyses

E: 28 children / 1 ECE<br><br>C: 28 children / same school as E.		SES not reported.	C: a traditional space with standard man-made equipment such as swings and climbing frame. It also had some natural elements like trees but much less than the natural playground.			
**Natural elements within ECE**						
Mårtensson et al (2009), Sweden [53].<br><br>E: 198 children / 11 ECE	Cross-sectional	Age: 5.26 (0.56 SD)<br><br>Gender: 113m / 85f<br><br>SES not reported	The outdoor settings of each preschool were dichotomized into ‘‘high-score’’ and ‘‘low-score’’ environments in analysis<br><br>The following were assessed:<br>A. Total outdoor area. 1= small (<2000 m^2), 2= medium (2000–6000 m^2), 3= large (46000 m^2)<br>B. Proportion of the area containing shrubbery, trees or hilly terrain: 1= little/non-existent, 2= <half of the area, 3= >half of the area<br>C. Integration between vegetation, open areas and play structures: 1= no integration. 2= either (a) Play structures adjacent to trees and shrubbery or integrated into areas, or (b) The open spaces are located in between play-areas and not in separate parts of the environment. 3= environments fulfilling both 2a and 2b above.<br><br>Outdoor environments were scored 1, 2 or 3 along three elements. The three scores of each environment were summed up and divided by 3, yielding an average score for each environment ranging from 1 to 3.	N/A	Cognitive	Nested mixed model
Sando (2019), Norway [50].<br><br>E: 80 children / 8 ECE	Cross-sectional	Age:3.5 (SD=0.5)<br><br>Gender: 41m/39f<br><br>SES not reported.	The places and materials in the playground were categorised into nature, pathways, open area and fixed functional equipment.	N/A	Social and emotional	A random intercept multilevel model

			Nature was coded in four of the institutions and ranged from large forest areas (1500 m^2) to smaller areas with trees and natural surfaces.			Covariates: age, gender
Söderström at al (2013), Sweden [55].<br><br>E: 172 children / 9 ECE	Cross-sectional	Presented per ECE<br>Age:<br>S1: 4.6 (1.0 SD)<br>S2. 4.1 (0.5 SD)<br>S3: 4.3 (0.7 SD)<br>S4: 4.4 (0.8 SD)<br>S5: 4.7 (0.8 SD)<br>S6: 4.6 (0.9 SD)<br>S7: 4.3 (0.9 SD)<br>S8: 4.6 (0.6 SD)<br>S9: 4.8 (0.7 SD)<br><br>Gender: % f<br><br>S1: 29%<br>S2. 41%<br>S3: 50 %<br>S4: 42%<br>S5: 50%<br>S6: 56%<br>S7: 61%<br>S8: 41%<br>S9: 63%	Outdoor Play Environment Categories (OPEC) scoring tool was used to assess playgrounds on (i) total outdoor area, (ii) amount of trees, shrubbery and hilly terrain and (iii) integration between vegetation, open areas and play structures, each component with a score range of 1–3 (high score = high quality).<br><br>The OPEC scores were then dichotomized (low OPEC value< 2, high OPEC value >2)	N/A	Social and emotional	ANOVA and MANOVA<br>Covariates:<br>Age, gender, birth<br>Weight, mother SES.
Zamani (2013), USA [37].<br><br>36 children / 1 ECE	Cross-sectional (mixed-methods – thesis)	Age: 4-5 years<br><br>Gender: 21M/15 F<br><br>SES not reported	Natural zone: wild landscape with non-structured green space (0.40 acres). The natural zone is rich in natural loose elements, such as leaves, twigs, dirt, stones and includes two looped and one straight pathways and boulders. The crawling equipment referred as the "green tube" is the sole manufactured element. This zone also includes three rope settings, tied to the trees.	N/A	Play	Chi square analysis

			Mixed zone: A widespread mixed outdoor environment of 0.48 acres referred as the "hill". The mixed zone has a moderate, downward slope from its entrance. There is rocking equipment, a linear pathway along the hill, a music wall with a stage, a set of six swings, a sand box, a gazebo, a stoned stone-lined swale without water, and two dramatic play settings. There is also a wood which includes a wooden platform, ropes, and musical instruments attached to the trees<br><br>Manufactured zone: a dramatic play setting (play house), a looped pathway, a composite play structure, a porch, a sand play setting (covered with a shade structure), bike sheds, bikes and scooters, storage (for storing toys and loose material), three gathering settings (benches and tables), a swing pergola, and a basketball loop. This zone also includes a transitional space between the indoors and outdoors. The manufactured zone has a smaller square footage (0.11 acres) compared to the other zones.			
**Garden-based intervention**						
Lillard (2016), USA [38].<br><br>E: 55 children / 1 ECE<br><br>Delay Gratification<br>E: 34 children	Uncontrolled before & after	Age: delay gratification= 4.16 years (9.9 months); Beery = 4.07 years (339.38 days)<br><br>Gender: 40m/51f (based on students who were assessed)	Gardening programme (not clearly described).	6 months	Cognitive	Repeated measures ANOVA

Visual motor integration<br>E: 39 children		SES not reported				
Park et al (2016), South Korea [64].<br><br>E: 336 children /12 ECE<br><br>Science investigation abilities and attitudes= 68 children	Uncontrolled before & after	Age: 5-7 years<br><br>Gender: 169m/167f<br><br>SES not reported.	The intervention consisted of horticultural activities that increase children's knowledge of seeds, soil, planting and harvesting etc. The intervention consisted of 24 sessions delivered once per week and lasted an average of 50 minutes per session	Intervention lasted 24 weeks. Outcomes were assessed one week prior to the intervention and one-week post intervention	Cognitive<br>Social and emotional	Paired samples t-test
Abbreviations: E= experimental; C= control; n= number; m=male; f= female; ECE = Early Childhood Education (includes preschools, day care, kindergarten etc.); SD= standard deviation; SE= standard error; SES= socioeconomic status; USD= US Dollars; GLM = General linear modelling.						

Table S2. Characteristics of included qualitative studies

**Author, year and country**	**Age (range or mean ± SD), sex (n or % m/f), SES.**	**Exposure and comparison**	**Research aims**	**Data collection method**	**Details of analysis**
**Nature-based ECE**					

Bjørgen (2016), Norway [51].<br><br>24 children / 1 ECE	Age: 3-5 years<br><br>Gender: 10m/14f<br><br>SES not reported.	Children played in the ECE outdoor play space for 3 hr/day, and each week would go on trips (1 or 2x) to natural environments.<br><br>The large outdoor area consists of outdoor toys (buckets, shovels, trucks, balls), swings, sandboxes, climbing racks, natural materials, small trees, a varied surface of grass, sand, asphalt, and small hills.<br><br>The destination for excursions in diverse natural landscape environment is approximately 300–700m from the centre. One type of natural environment was open fields suitable for tobogganing, running and playing on skis. Another natural environment consisted of woods. Trips were made to the natural environments all year round.	What is the relation between environmental affordances and PA levels among 3–5 year olds?	Observations were made with video recording the different seasons of the year for 20 days, 10 days on trips in a natural environment and 10 days in the centres play space. A total of 50 h of direct observation was conducted.<br><br>Coding of the physical activity levels of children was assessed and adapted using the Observational System for Recording Physical Activity in Children-Preschool Version (OSRAC-P) manual.	Thematic analysis - the first phases of coding were assessing and identifying the children's level of PA in different play situations. Figures were used as an analytical tool helped to discern patterns, differences and similarities in the data material, which laid foundations for the qualitative analysis of the affordances. Thereafter themes of affordances are identified within the data. The theory of affordances and criteria from the 7Sc were used in the analysis process.
Dowdell et al (2011), Australia [44].<br><br>E: 6 children / 1 ECE<br><br>C: E: 6 children / 1 ECE	Age: 2-6 years<br><br>Gender: 6m/6f<br><br>SES not reported.	E: Has an emphasis on nature and sustainable education. The space is large and consists of sandpit, fairy garden, play equipment, grass area and vegetable garden.<br><br>C: Located in a warehouse this centre has an entirely artificial indoor play area. It consists of a bike track, home corner (playhouse etc), climbing structures, quiet play area, sandpit and obstacle course.	How are children's play behaviours and social interactions influenced by the opportunities and materials present in their outdoor play environment?	Play behaviours were recorded using a behaviour mapping schedule. Each child was observed individually and every 10 seconds an observation based on social interaction and play behaviour was recorded.	Once all the observations were made for each child at each centre they were then tallied up.<br><br>Play behaviours were then categorised into four different groups: social activities, cognitive activities, physical and motor skill activities and other activities.

Liu (2020), USA [39].<br><br>Nature interaction:<br>E: 29 children / 1 ECE<br><br>C: 26/ 1 ECE<br><br>Restorative experiences:<br>E: 10 children / 1 ECE<br><br>C: 9 children/ 1 ECE	Age: 4-5 years<br><br>Gender: 30m/ 25f<br><br>SES: E: 48,000 US (household income); C: 59,000 (household income) of children attending each centre	E: contains high levels of nature with a variety of perceived affordances. Outdoor time = 1.5 hours/day. 32 types (categories- vegetation (tress, shrubs, flowers, grasses), natural ground surface (wood chips, meadow, multipurpose lawns), natural materials, natural play structures (e.g. wood, stick, water, sand logs, ice, leaves), animals, experiential elements (rain, snow, sky view, light, air) of natural elements and play settings and 4 types of non-nature-based play settings (concrete track, bicycles, concrete hall, concrete sq.) were identified<br><br>C: low levels of nature and perceived affordances. Outdoor time = 1.5 hours/day. 13 types of natural elements and 11 (vegetation, natural ground, animals) types of non-nature-based play settings (examples include: play structure, playhouse, outdoor kitchen, bicycles) were identified.	How does the designed nature-based outdoor play environment in ECE impact children's interaction with natural elements?<br><br>How does the designed nature-based outdoor play environment in ECE impact children's restorative experience?	RQ 1. Field observation, behaviour mapping, semi-structured interview with teachers.<br><br>RQ2. Field observation, structured Interview with children, semi-structured interview with teachers.	Content analysis was used for: children's frequent play locations, types of play behaviors, frequency and diversity of different ways of interaction with natural elements, as well as restorative experience from semi-structured interviews with teacher and structured interview with children.<br><br>Themes (coding categories) were drawn from the theoretical framework. Specifically, children's types of play behaviors and their ways of interacting with natural elements were coded using function taxonomy of affordance (Heft, 1988; Kyttä, 2002) and Gibson's affordance theory.
Maynard et al (2013), Wales, UK [58].<br><br>48 children / 8 ECE	Age: 4-7 years<br><br>Gender: 24m/24f<br><br>SES not reported.	Educators introduced child-initiated learning in the outdoor environments. The kinds of activities varied and incorporated free play with natural resources (e.g. ECE A, F and H); growing vegetables (ECE C); (ECE B); and more structured investigations – for example, of snails (ECE D), air/wind (ECE E) and flight (School G).<br><br>All the teachers had access to a small tarmac yard or grassed area. These were	To explore these perceived differences as well as teachers' perceptions of 'underachievem ent'.	Researcher visited teachers three times to undertake individual semi-structured interviews. Interviews were audio recorded and field notes at each interview.<br><br>Teachers also provided case studies of each student	Interviews were transcribed using Nvivo8. A thematic analysis approach was used where data were analysed in three ways with increasing depth:<br>1. perceived difficulties of children<br>2. case studies<br>3. theoretic issues related to "place and space"

		seen by the teachers as ‘outdoor classrooms’ and used for painting, sand and water play, construction activities etc. The teachers also had access to some additional outdoor space – playing fields, vegetable gardens or common land. 3 ECE settings (A, G and H) had extensive outdoor environments incorporating different types of play equipment or natural features such as a willow tunnel and pond.			
Sandseter (2009), Norway [52].<br><br>29 children from both experimental and control groups<br><br>E: 1 ECE<br><br>C: 1 ECE	Age: 4-5 years<br><br>Gender: 21f/8m<br><br>SES not reported.	E: Located in a forest with no fixed play equipment and fencing and children spent most of their time outdoors.<br><br>C: fixed equipment, such as swings, climbing tower, play hut and a few trees.	To explore affordances for risky play in two different play environments: an ordinary ECE playground and a nature playground.	7 days were spent on each of the ECE playgrounds. Video recordings and field notes of risky play situations were collected based on categories of risky play; a) great heights, b) high speed c) dangerous tools, d) dangerous elements, e) rough-and-tumble play, f) where the children can disappear/get lost. Both the children’s play and the staff’s supervision were observed. The field notes and the video recordings were transcribed into an electronic word file.<br><br>12 children in the ordinary preschool and 11 children in the nature and outdoor preschool participated in a one-to-one qualitative interview with the researcher. Each interview was approximately 20-	A content analysis was performed on the data. The analysis was theory-driven. Firstly, each of the play environments’ potential affordances for risky play, as categorized by Sandseter (2007), were analysed in relation to the most relevant affordance categories to evaluate their potential affordances for risky play. Secondly, the transcriptions of the video observations, field notes, and interviews were examined to determine the types of risky play children engaged in within different environments.<br>Thirdly, the observations and the interviews were analysed to determine the degree to which children experienced mobility

				30 minutes and was recorded on audiotape. The interviews were semi-structured, using an interview guide list of questions and issues. The interview guide was based on the six categories of risky play and aimed to explore the types of risky play that the children engaged in within the different play environments and whether the staff constrained or intervened in their actions. Upon completion of the interviews, the audiotapes were professionally transcribed verbatim into an electronic word file.	license while engaging in risky play. The transcriptions of the video observations were examined to determine the extent to which, and in which situations, the staff had children under surveillance while they engaged in risky play or was taking initiative to or constrained risky play.
Streelasky (2019), Canada [49].<br><br>15 children / 1 ECE	Age: 5-6 years<br><br>Gender not reported.<br><br>SES not reported.	The ECE setting had an outdoor, nature-based focus where children spent afternoons in the forested area. The teacher who was involved in an Outdoor Environmental Leadership Programme engaged the students in an integrated learning approach where key curriculum areas were addressed (e.g. language arts, social studies, science and physical education). Children also had time to freely explore the forest.	What learning experiences do kindergarten children value at school? and what modes are they choosing to express and represent their valued school learning experiences?	Qualitative interpretative approach involving (i) group discussions, (ii) participant observations, (iii) anecdotal notes, (iv) artefact collection and (v) individual semi-structured interviews (children's narratives).	Data were analysed and grouped into themes.<br><br>Image based analysis was used to develop deeper understanding of children's interests and knowledge.<br><br>Thematic analysis was used to gain insight into children's practices which followed 6 phases: (i) familiarising oneself with the data and identifying items of potential interest, (ii) generating initial codes, (iii) searching for themes,

					(iv) reviewing potential themes, (v) defining and naming themes and (vi) reporting the themes.
**ECE natural playgrounds**					
Herrington & Studtmann (1998), USA [40].<br><br>36 children / 1 ECE (2 “labs”)	Age: 2-6 years<br><br>Gender: 16m/20f<br><br>SES not reported.	Pre-modification:<br>Lab A: consisted of a patio area, grass lawn, play structures, swing set, doll house, trees and vegetation.<br><br>Lab C consisted of a porch area, grass lawn, play areas, swing set, trees and vegetation.<br><br>Post-modification:<br>Playground were naturalised with increased natural elements: ice sculptures, wind chimes, canopy, chalk, buckets, playhouse, water pay, vegetation and trees were added to the labs.<br><br>Lab A received more natural elements than lab C but both were more natural post intervention.	What natural materials and conditions of the outdoor environment can contribute to the development of young children ranging from 2 to 6 years old?	Phase 1: sequence sampling of children during free-play. Children were video-taped interacting with the site for 1 month. Once the modifications were made, data collection began a week later.<br><br>Data collection involved video-taping, sound recording, and field notes.<br><br>Videotaping involved following a child for 20 minutes as they moved throughout the yard in free play. Voice recordings of the children were made of one of the two selected children from each Lab. Voice recordings were transcribed into text documents. Field notes (weather, teacher and children present, anecdotal observations etc.) were made daily by researchers. Notes were recorded by researchers on a pre-printed notation	20 hours of videotapes were analysed. During analysis, notes were made. For Phase 1 the notes were: (1) interaction with an intervention (2) duration of interaction (3) children's behavioural modification made between pre and post intervention (4) children's movement changes made between pre and post intervention.<br><br>For Phase 2 the criteria were: (1) which children were engaged in the intervention; (2) how many children were engaged (3) the duration and nature of their engagement with the intervention (4) how behavior and paths of movement changed between pre and post intervention.<br><br>Video clips were selected that illustrated the notes. These clips were put

				sheet that displayed a plan view of both yards.<br><br>Phase 2: Video documentation and anecdotal notes were employed to record event sampling. Event sampling allowed subjects to be taped if they interacted with the plant interventions. The specific intervention sites were recorded on a rotating basis. Children were video-taped using the same schedule as in Phase 1 and fieldnotes were made in the same manner as in Phase I	together on one VCR tape using a television and VCR recorder. The conversations of the children participating in Phase 1 were transcribed at 10 second intervals. The anecdotal notes were reviewed and complied.
Puhakka et al (2019), Finland [61].<br><br>12-24 children (not clear) /<br>6 ECE	Age: 3-5 years<br><br>Gender not reported.<br><br>SES not reported.	Playground yards were transformed through enhancing the biodiversity by incorporating more greenspace and vegetation. For example, replacing areas covered in gravel with forest floor.<br><br>Children spent time outdoors every day (0.5–2 h in the morning and in the afternoon) as well as participating in teacher led activities 4-5 days/ week.	Does biodiversity exposure and greening playgrounds affect 3–5 years-old children's physical activity and play, their environmental relationships, and their well-being in the urban environment in Finland.	Educators and child nurses completed interviews and surveys respectively. 49 parents completed surveys.<br><br>Surveys were completed one month after the playground was modified. Surveys included both structured and open ended questions which related to children's play activities, and enthusiasm. Interviews with parents focussed on children perception of modifications. The educator thematic interviews focused on possible changes in children's play and other activities in	Interviews were recorded and transcribed verbatim. Survey and interview data were analysed using qualitative content analysis to identify different affordances. The affordances were then classified into 6 themes which emerged from analysis and coding.<br><br>How these affordances supported children's relationship with the modified playground were then mapped.<br><br>Finally, these two elements

				the yard, in children's and educators interest in and knowledge of nature, their well-being, attitudes towards outdoor activities, and practices and atmosphere in the ECE setting	were brought together to form three perspectives.
Wishart et al (2019), Australia [45].<br><br>75 children / 1 ECE	Age:4-5 years<br><br>Gender not reported.<br><br>SES not reported.	The two playgrounds were located on different sides of the building, each extending to the back of the building where a connecting gate was sometimes opened to allow free-flow of children between the two spaces.<br><br>E: Traditional equipment was replaced with terraces, inclines, logs and rocks designed to afford physical activities and gross motor skills such as climbing and balancing. other elements included: Natural gardens with fruit trees; herb garden and small plants; logs; stepping-stones; log enclosure; small tree forest; sandpit with pebbles and medium-size rocks.<br><br>C: standard equipment: slide, ladders, swings, climbing frames, sand-pit, surfaces open area. This area also included a grass area, veg garden, trees and shrubs.	Does the naturalised design of the new space provide equivalent actualisable affordances for different types of physical activity to those provided by the more traditional playspace, with its conventional equipment and resources	Behaviour mapping using a time-sampling observation tool. Observations were conducted between 10:30–15:30 during sessions. The two playscapes were divided into zones and children were observed in 3 minute cycles. For each observation, the tool also noted: number of boys and girls (no further count of children was taken); presence of educators; whether play was solitary or group; location and general contextual information.<br><br>40 observations in the naturalised space and 42 observations in the traditional space were made.	Behaviour mapping tracked the incidence of different categories of movement across different areas of the two playscapes, to investigate if different categories of movement were more likely to occur in specific areas or in relation to specific features.
**Natural elements within ECE**					
Zamani (2015), USA [37].	Age: 4-5 years	See quantitative study characteristics table.	How does an outdoor learning	1. Photo preference - researcher captured photos	1. Used with transcribed child interviews and then

36 children / 1 ECE	Gender: 21M/15 F<br><br>SES not reported.		environment with natural features can stimulate children’s cognitive play behaviors	based on particular behavior settings or elements of the outdoor environment. The photos represented particular spaces in which children engaged in certain behaviors. The researcher used photo preference to ask children to select their preferred outdoor settings and elements and explain about their play.<br><br>2. Drawings from children - The researcher asked children to draw their favourite outdoor play spaces as a means for the researcher to evaluate each setting’s cognitive play affordances and the elements children enjoyed.<br><br>3. Structured interviews with children - Interview questions aimed toward understanding children’s choice of photos, drawings, and opinions of the outdoor learning environment.<br><br>4. structured interviews with teachers - to understand the teachers’ perspectives toward the outdoor environment and children’s daily interactions. The interview questions (6) prompted teachers to discuss	coded these into different cognitive play behaviours. The photos were used to understand child's explanations.<br><br>2. The analysis of the drawings included three stages. In the first stage, the researcher quantified all 22 drawings by coding their visual features; The drawing codes established the element or behavior setting types depicted in the image; The researcher further evaluated the drawings on the frequency that certain settings or elements appeared<br><br>3. Interviews recorded and transcribed and then grouped by themes<br><br>4. transcribed and then grouped into themes related to teachers view on curriculum, outdoor learning environment, value of children's play, what children prefer, cognitive play affordances.

				the play opportunities the different zones provided for children. The following section explains the protocols regarding each of the described methods.	
Abbreviations: E= experimental; C= control; n= number; m=male; f= female; ECE = Early Childhood Education (includes preschools, day care, kindergarten etc.); SES= socioeconomic status; PA= physical activity.					

Additional file S5. Findings per eligible study
Quantitative

Social and Emotional

Table S3. Nature-based ECE on social and emotional outcomes								
**Study details** (Author, year and country)<br><br>**Sample size** (n of children / n ECE settings for exp and con)	**Study Design**	**Outcome and measurement**	**Units**	**Baseline or one time point (cross-sectional)**	**Follow-up (if applicable) or mean difference**	**Summary of Findings**	**Effect Direction**	**Quality Rating**
**Nature-based ECE**								
Agostini et al (2018), Italy [59].	Controlled Before & After study	Kuno Beller Developmental Tables completed by educators which assesses		T1 (Jan 2014)<br><br>E:11.18 (1.09 SD)<br>C:10.24 (1.14 SD)	T4 (May 2015)<br><br>12.96 (0.94 SD)<br>12.86 (0.94 SD)	There was significant time x group interaction on children's social and	▲	Weak

E: 41 children / 7 teachers / 1 school<br><br>C: 52 children / 13 teachers / 1 school		development in 8 developmental areas: Body Function, Awareness of the Surrounding Environment, Social and Emotional Development, Play, Language, Cognitive Development, Gross and Fine Motor Skills.	Social and emotional development		p= 0.000; $\eta p2= 0.38$	emotional development.<br><br>There were no significant differences between groups at T4.		
Cooper (2018), United Kingdom (England) [56].<br><br>E: 13 children<br><br>C: 11 children<br><br>Children from the same school	Controlled before & after study	The Devereux Early Childhood Assessment for Pre-schoolers, Second Edition (DECA-P2) consists of 38 items on a 5-point likert scale. The assessment measures protective factors and screen for behavioural concerns. The protective factors are divided into 3 subscales: initiative, self-regulation and attachment/ relationships which form an overall measure of social and emotional wellbeing when combined.<br><br>Parent and teachers completed the form and they were asked to reflect on the child's	Attachment / relationships<br><br>Self-regulation<br><br>Initiative<br><br>Social and emotional wellbeing<br><br>(median and range)	E: 23 (13)<br><br>C: 25 (15)<br><br>Presented in cognitive domain<br><br>E: 21 (14)<br><br>C: 20 (12)<br><br>E: 69 (40)<br><br>C: 71 (39)	27 (11); Z=2.82 p=0.005<br><br>31 (17); Z=2.61 p=0.009<br><br>**U=32.0 p=0.058**<br><br>Presented in cognitive domain<br><br>26 (13); Z=2.41 p=0.016<br><br>29 (16); 2.63 p=0.009<br><br>**U=40.5 p=0.187**<br><br>76 (32); Z=2.49 p=0.013<br><br>83 (48); Z=2.49 p=0.013	No statistically significant between-group differences at T2 for attachment/ relationships, initiative, and social and emotional wellbeing	▼<br><br>▼<br><br>▼	Weak

		behaviour for the previous 2 weeks.			**U=42.0 p=0.224**			
Cordiano et al (2019), USA [26].<br>E: 12 children / 1 ECE class.<br>C: 14 children / 1 class.<br>Children from the same school.	Controlled before & after	Preschool and Kindergarten Behavior Scales, Second Edition (PKBS-2) is a 76-item behavior rating instrument which assesses social skills and behavioural problems. The Social Skills scale assess the dimensions of Social Cooperation, Social Interaction, and Social Independence. The Problem Behavior scale assesses the dimensions of Externalizing Problems and Internalizing Problems	Social skills<br><br>Teacher<br><br><br><br><br><br><br><br><br><br><br><br><br>Parent	T1 - baseline<br><br>E:101.92 (11.69 SD)<br>C: 110.07 (7.41 SD)<br><br><br><br><br><br><br><br><br><br>E:102.20 (15.51 SD ;<br>C: 104.00 (7.29 SD)	T3 - endpoint<br><br>106.21 (13.34 SD)<br>112.96 (6.29 SD)<br><br>Within-group: p= non-sig, $\eta 2p$= 0.01<br>Between group: F=1.98, $\eta 2p$= 0.08, p> 0.05<br><br>108.40 (12.67 SD)<br>128.73 (64.96 SD)<br>Within-group: p= non-sig, $\eta 2p$= 0 .08<br>Between group: F= 0.87, $\eta 2p$= 0.05, p> 0.05	Small effect for between group<br><br><br><br><br><br><br><br><br><br><br>Small effect for between group	▼<br><br><br><br><br><br><br><br><br><br><br><br>▼	Weak
			Behavioural problems<br><br>Teacher	E: 91.58 (9.14 SD)<br>C: 82.46 (6.39 SD)	89.96 (12.26 SD)<br><br>83.93 (5.03 SD)<br><br>Within-group: p= non-sig, $\eta 2p$= 0.01<br>Between group:	Moderate effect for between group	▼	

					F=4.81, $\eta 2p$= 0.17, p<0.05			
			Parent	E: 97.00 (21.12 SD)<br>C: 101.10 (13.16 SD)	92.67 (16.52 SD)<br><br>95.20 (9.94 SD);<br><br>Within-group: p= non-sig, $\eta 2p$= 0.21<br><br>Between group: F= 0.15, $\eta 2p$= 0.01, p>0.05	No effect for between group	▲	
Müller et al (2017), Canada [47].<br><br>E: 43 children / 1 nature-kindergarten<br><br>C: 45 children / 1 traditional kindergarten	Controlled before & after study	Social Skills Rating Scale (SSRS) completed by parents and teachers. This assesses the following social skills: cooperation, assertiveness, social responsibility and self-control and items assessing psychological health (internalising and externalising behaviour). Questionnaires were completed by teachers and parents. They were asked to indicate how often a behavior occurred (never, sometimes, very often).	Teachers<br><br>Assertiveness	E:17.15 (0.57 SE)<br>C:12.40 (0.55 SE)	19.16 (0.47 SE)<br>12.86 (0.45 SE)<br>p= 0.00, η2= 0.34	At post-test there was a large and significant effect.	▲	Weak
			Cooperation	E:17.14 (0.52 SE)<br>C:15.00 (0.49 SE)	18.63 (0.45 SE)<br>15.25 (0.43 SE)<br>p= 0.00 η2= 0.20	As above.	▲	
			Self-control	Presented in cognitive domain.	Presented in cognitive domain.			
			Externalizing Behavior:	E: 2.63 (0.48 SE)<br>C: 1.91 (0.47 SE)	2.05 (0.43 SE)<br>1.98 (0.41 SE)<br>p= 0.11, η2= 0.03	At post-test there was a small and non-significant effect.	▼	
			Internalizing Behavior	E: 0.96 (0.16 SE)<br>C: 0.36 (0.15 SE)	0.20 (0.11 SE)<br>0.41 (0.10 SE)<br>p= 0.04, η2= 0.05	At post-test there was a small and significant effect.	▲	
			Parent					

			Assertiveness	E:15.27 (0.43 SE) C:15.31 (0.62 SE)	16.24 (0.42 SE) 14.75 (0.60 SE) p= 0.01, η2= 0.13	At post-test there was a moderate and significant effect.	▲	
			Social Responsibility	E:11.58 (0.48 SE) C:10.50 (0.67 SE)	13.10 (0.44 SE) 11.06 (0.61 SE) p= 0.03, η2= 0.11	As above.	▲	
			Cooperation	E:12.76 (0.37 SE) C:12.00 (0.52 SE)	13.18 (0.36 SE) 11.75 (0.52 SE) p= 0.06, η2= 0.08	At post-test there was a moderate but non-significant effect.	▲	
			Self-control	Presented in cognitive domain.	Presented in cognitive domain.			
			Externalizing Behavior:	E: 3.67 (0.38 SE) C: 3.79 (0.50 SE)	3.06 (0.36 SE) 3.63 (0.47 SE) p= 0.25, η2= 0.03	As above.	▲	
			Internalizing Behavior	E: 1.17 (0.17 SE) C: 0.79 (0.23 SE)	0.94 (0.17 SE) 0.90 (0.23 SE) p= 0.68, η2= 0.00	At post-test there was a non-significant effect.	▼	
Ernst et al (2019) & Ernst & Burcak (2019), USA [29, 30]. E: 78 children / 4 ECE	Uncontrolled Before & After study	Resilience Devereux Early Childhood Assessment for Preschoolers, Second Edition (DECAP2) - Parents and teachers evaluate 27 positive behaviors, which form 3 subscales: initiative, self-regulation, and attachment. Three subscales were converted to standard	Teacher: Total protective factors	E:54.54 (5.95 SD)	57.71 (7.87 SD), p=0.01	Significant improvements in total protective factors and initiative in the nature preschool from baseline to follow-up. No significant improvements in attachment scores.	▲	Weak
			Initiative:	E:52.74 (7.98 SD)	56.93 (8.55 SD), p= 0.01		▲	
			Self-regulation:	presented in cognitive domain.	presented in cognitive domain.			
			Attachment:	E:55.26 (6.91 SD)	57.21 (7.45 SD)		▲	

		scores (T-scores) with a mean of 50 and SD of 10.	Parent					
			Total protective factors	E:50.21 (7.62 SD)	53.13 (8.81 SD), p = 0.01	Significant improvements in in the total protective factors, and initiative in the nature preschool from baseline to follow-up. No significant improvements in attachment scores.	▲	
			Initiative	E:49.84 (8.45 SD)	53.63 (8.17 SD), p= 0.01		▲	
			Self-regulation:	presented in cognitive domain.	presented in cognitive domain.			
			Attachment:	E:51.64 (7.24 SD)	51.39 (9.93 SD)		▲	
Fyfe-Johnson et al (2019), USA [33].<br><br>E: 20 children / 1 ECE<br><br>C: 13 children (waitlist control or 2-hour nature-based, outdoor enrichment class provided by experimental ECE	Controlled cross-sectional	Child behaviour<br>SDQ: 25-items consisting of 5 domains: emotional problems, conduct problems, hyperactivity/ inattention, peer relationship problems, and prosocial behavior.<br>Parents rated their child on a scale of 0 to 2 per question (0=not true; 1=somewhat true; 2=certainly true). Overall score was calculated (sum of all domain scores except prosocial behavior; overall score range: 0-40). Prosocial was scored separately.			Mean diff			Weak
			Overall Score	E: 6.55 (4.35 SD)<br>C: 7.51 (4.23 SD)	-0.95 (95% CI: -4.39, 2.49)	Children in the nature ECE did not differ in behavioural scores compared to the control.	▲	
			Emotional problems	E: 1.20 (1.67 SD)<br>C: 1.00 (0.95 SD)	0.2 (95% CI: -0.82, 1.22)		▼	
			Conduct problems	E: 1.63 (1.54 SD)<br>C: 1.83 (1.59 SD)	-0.23 (95% CI: -1.49, 1.03)		▲	
			Hyperactivity/ inattention	presented in cognitive domain.	presented in cognitive domain.			
			Peer relationship problems	E: 1.05 (0.94 SD),<br>C: 1.08 (1.24 SD)	-0.03 (95% CI: -0.95, 0.88)		▲	
			Prosocial behavior	E: 8.15 (1.57 SD),<br>C: 7.83 (1.59 SD)	0.32 (95% CI: -0.95, 1.59)		▲	

Ernst (2014), USA [28].<br><br>E: 46 educators	Cross-sectional	Questionnaire (not described) on importance of natural outdoor settings on children's cognitive, social, and physical development and their appreciation for the environment. Responses were provided on a five-point scale, ranging from one (strongly disagree) to five (strongly agree)	Social development (1-5)	4.43 (1.31 SD), r= 0.05		There was no association between frequency of nature experiences and belief regarding importance of outdoor settings for social development.		Weak
**ECE natural playgrounds**								
Brussoni et al (2017), Canada [48].<br><br>E: 48 children / 2 childcare centres	Uncontrolled before & after (mixed methods)	Sociometric status was determined by rating how "dominant or influential" and "popular" each child is with peers	Sociometric:<br><br>Dominance		Centre A= 3.42<br>Centre B= 2.70	Mean sociometric scores remained stable over time.	▲	Moderate
			Acceptance		Centre A= 3.44<br>Centre B= 3.25		▲	
		Strengths and difficulties questionnaire (SDQ)- 25 items that measure emotional symptoms, conduct problems, hyperactivity, peer relationships, and prosocial behaviour.	Strengths and difficulties (median)	2.3	2.0;<br>z= -2.10, p= 0.036	There was a significant decrease in the SDQ peer problems scale.<br><br>No other scores differed significantly (not reported).	▲	

		Preschool social behaviours skill (PSBS-T) - 19 items assessing relational aggression, overt aggression, depressed affect.	Social behaviour (median)	6.0	3.0<br>z= -2.24, p= 0.03	There was significant decrease in The PSBS depression score.<br><br>No other scores differed significantly (not reported).	▲	
Cosco et al (2014), USA [35].<br><br>E: not clear / 27 centres.	Uncontrolled Before & After study	Social interactions<br><br>Observational behaviour mapping was conducted. Location of children, gender, PA level, social interactions (alone, pair, group), teacher interactions (not present, positive, custodial, negative) were recorded by observers and entered into a handheld computer.	Custodial (i.e tying shoe laces, offering water) teacher-child interaction		-0.156, B=-0.095), p< 0.05	At follow- up, observations highlighted significantly less custodial teacher-child interactions, more negative teacher-child interactions, less positive teacher-child interactions and less children with another child or in a group:	▼	Weak
			Negative teacher-child interaction		0.030, B= - 034, p< 0.05		▼	
			No teacher present		0.082, B= - 0.002, non-sig		▼	
			Positive teacher-child interaction		- 0.064, B= - 0.088, p< 0.05		▼	
			Child is alone		- 0.195, B= not estimated			
			Child is with one other child		- 0.034, B= - 0.031, p< 0.05		▼	
			Child is in group		- 0.168, B= - 0.113, p< 0.05		▼	
Carrus (2012), Italy [60].	Cross-sectional	Social interactions	small group play	t (9)= 2.36; p= 0.02)		There was a significantly higher	▲	Weak

E: 16 children / 1 ECE		Frequency of small group play, self-organised play, direct interventions by educators, boredom feelings episodes were observed.<br><br>Trained observers recorded and coded these on a six-step scale, ranging from 0= never to 5 = always.	self-organised play<br><br>direct interventions by educators<br><br>boredom feelings episodes	t (9)= 2.36; p= 0.03<br><br>t (9) = -1.42; p = 0.09<br><br>t (9) = -1.48; p= 0.09		frequency of small group play and self-organised play in the external green space compared to the internal space. There was not a significantly lower frequency of direct interventions by educators and of boredom feelings episodes	▲<br><br>▲<br><br>▲	
		Stress<br><br>Frequency of dispute-resolution interventions by educators, crying episodes and capacity of being quickly comforted in case of crying were observed.<br><br>Trained observers recorded and coded these on a six-step scale, ranging from 0= never to 5 = always.	Dispute-resolution interventions by educators<br><br>Crying episodes<br><br>Capacity of being quickly comforted in case of crying	F (1, 9) = 7,63; p= 0.022; eta square = 0.46<br><br>F (1, 9) = 4,46; p= 0.064; eta square = 0.33<br><br>F (1, 9) = 9,17; p = 0.014; eta square = 0.50		There was a significant 2- way interaction for frequency of dispute resolution interventions by educators and capacity of being quickly comforted in case of crying, but not frequency of crying episodes.	▲<br><br>▲<br><br>▲	
**Natural elements within ECE**								
Sando (2019), Norway [50].<br><br>E: 80 children / 8 ECE	Cross-sectional	Emotional wellbeing<br><br>Leuven Well-Being Scale which assesses children's emotional wellbeing. This is an observational assessment where	Emotional Wellbeing (1-5)	Well-being 3.6 (0.6 SD), (regression coefficient = 0.004, p=< 0.05)		Nature was a statistically significant predictor of emotional wellbeing	▲	Weak

		children are scored on a scale from 1 to 5. 1= clear signs of discomfort (screaming, anger, sadness) and 5= happy, relaxed.							
Söderström at al (2013), Sweden [55].<br><br>E: 172 children / 9 ECE	Cross-sectional	Stress<br>The Salivette®kit (Sarstedt, Numbrecht, Germany). Children were asked to chew a swab for 1 min once in the mid-morning (AM cortisol, 9–10 am) and again the afternoon (PM cortisol, 1 –2 pm). The difference between PM cortisol and AM cortisol was calculated. A positive value implied a rise in PM cortisol level suggesting increased stress.	Stress (PM = AM cortisol)	Low OPEC: -0.4 (1.3 SD)<br><br>High OPEC: -4.4 (1.9 SD)<br><br>p= 0.41			Outdoor environment quality was associated with reduced stress.	▲	Weak
**Garden-based intervention**									
Park et al (2016), South Korea [64].<br><br>E: 336 children /12 ECE<br><br>Prosocial behaviour: 133 children	Uncontrolled before & after	The revised prosocial behavior questionnaire by Lee (1996) was used. This consists of 20 questions on 4 subscales: helping, sharing, cooperation and kindness. Answers are given on a three-point likert scale (agree, neutral,	Emotional intelligence (1-5):<br><br>Utilization of emotions<br><br>Recognition and consideration	 <br>3.35 ± 0.83<br><br>3.36 ± 0.59	 <br>4.01 ± 0.88, p=0.000<br><br>3.79 ± 0.68, p=0.000		Emotional intelligence: There was significant improvements in emotional intelligence subcategories from baseline to follow-up	▲	Weak

Emotional intelligence: 135 children		disagree. Teachers completed this questionnaire based on their daily observations. Higher scores indicate a more positive behaviour.	of others' emotions					
			Recognition and expression of own emotions	3.86 ± 0.73	4.30 ± 0.63, p=0.000			
			Emotional regulation and impulse control	3.62 ± 0.65	4.11 ± 0.81, p=0.000			
			Relationships with teachers	3.77 ± 0.90	4.19 ± 0.71, p=0.000			
			Relationships with peers	3.73 ± 0.92	4.09 ± 0.84, p=0.000			
		The emotional intelligence questionnaire consisted of 50 questions on a five-point likert scale (strongly agree - strongly disagree) which was completed by teachers. Higher scores indicate a more positive behaviour.	Prosocial behaviour (1-3).			Prosocial behaviour: There was significant improvements in prosocial behaviour subcategories from baseline to follow-up.	▲	
			Helping	2.37 ± 0.46	2.57 ± 0.43, p = 0.000			
			Sharing	2.53 ± 0.41	2.66 ± 0.36, p= 0.001			
			Cooperation	2.42 ± 0.43	2.66 ± 0.38, p= 0.000			
			Kindness	2.30 ± 0.38	2.55 ± 0.40, p= 0.000			

Abbreviations: E= experimental; C= control; n= number; ECE = Early Childhood Education (includes preschools, day care, kindergarten etc.); SD= standard deviation; SE= standard error; CI= confidence intervals.

Effect direction explained:
▲: positive health impact

►: no change/ conflicting findings
▼: negative health impact
▲: positive health impact and statistical significance ($p<0.05$)
▼: negative health impact and statistical significance ($p<0.05$)
No arrow: no inferential statistics reported

Controlled before & after studies – difference between experimental and control group at follow-up (unless stated) or difference in change between experimental or control group. Uncontrolled before & after studies – change since baseline (unless stated). Controlled cross sectional – difference between experimental and control (unless stated). Cross-sectional – positive, negative or no association.

Table S4. Nature-based ECE on play behaviour

**Study details** (Author, year and country)<br><br>**Sample size** (n of children / n ECE settings for exp and con)	**Study Design**	**Outcome and measurement**	**Units**	**Baseline or one time point (cross-sectional)**	**Follow-up (if applicable) or mean difference**	**Summary of Findings**	**Effect Direction**	**Quality Rating**
**Nature-based ECE**								
Agostini et al (2018), Italy [59].<br><br>E: 41 children / 7 teachers / 1 school<br><br>C: 52 children / 13 teachers / 1 school	Controlled Before & After study	Kuno Beller Developmental Tables completed by educators which assesses development in 8 developmental areas: Body Function, Awareness of the Surrounding Environment, Social and Emotional Development, Play, Language, Cognitive Development, Gross and Fine Motor Skills.	Play (mean and SD)	T1 (Jan 2014)<br><br>E:11.26 (1.08 SD)<br>C: 9.89 (1.22 SD)	T4 (May 2015)<br><br>13.15 (0.99 SD)<br>12.78 (1.14 SD)<br>$p= 0.00$;<br>$\eta p2= 0.41$	There was a significant time x group interaction on children's play.<br><br>There were no significant differences between groups at T4.	▲	Weak

Cordiano et al (2019), USA [26].<br>E: 12 children / 1 ECE class.<br><br>C: 14 children / 1 class.<br><br>Children from the same school.	Controlled before & after study	Play Interaction, Play Disruption, and Play Disconnection<br><br>Assessed using the Penn Interactive Peer Play Scale (PIPPS), which is a 32-item behaviour rating instrument assessing aspects of children's peer play behaviours.<br><br>Pretend Play rating consisted of 5 questions on a 5 point likert scale to assess children's imagination in play, use of make-believe, enjoyment of play, amount of emotion expressed in play, and use of make-believe in dramatic play.	Teacher<br><br>Play interaction	T1 - baseline<br><br>E:49.46 (6.99 SD)<br>C:54.96 (2.64 SD)	T3 - endpoint<br><br>54.69 (5.07 SD)<br>55.82 (2.76 SD)<br>Within group: $p<0.01$, η^2p= 0.26<br>Between group: (F=2.70, η2p= 0.11, $p>0.05$)	Small effect for between group	▼	Weak
			Pretend play	E:15.18 (1.66 SD)<br>C:18.21 (2.12 SD)	23.45 (2.12 SD)<br>18.86 (3.35 SD)<br>Within group: $p<0.01$ η2p= 0.29<br>Between group: F=0.00, η2p= 0.00, $p>0.05$	No effect for between group	▲	
			Play disruption	E:50.38 (5.96SD)<br>C:43.69 (6.43 SD)	47.71 (7.26 SD)<br>38.31 (5.53 SD)<br>Within group: non-sig, η2p= 0.06<br>Between group: F=17.64, η2p= 0.45, $p<0.001$	Large effect for between group	▼	
			Play disconnection	E:52.13 (7.34 SD)<br>C:43.71 (5.63 SD)	45.75 (9.28 SD)<br>40.14 (4.69 SD)<br>Within group non-sig, η2p= 0.08<br>Between group: F=14.59, η2p= 0.39, $p<0.01$	Large effect for between group	▼	

			Parent<br><br>Play interaction<br><br><br><br><br>Pretend play<br><br><br><br><br>Play disruption<br><br><br><br><br>Play disconnection	 <br>E:46.90 (6.72 SD)<br>C:48.00 (7.00 SD)<br><br><br><br>E:20.90 (3.54 SD)<br>C:21.80 (3.58 SD)<br><br><br><br>E:49.11 (9.21 SD)<br>C:50.00 (3.81 SD)<br><br><br>E:49.63 (11.20 SD)<br>C:50.33 (8.54 SD)	 <br>51.30 (7.46 SD)<br>51.22 (9.91 SD)<br>non-sig,<br>$\eta 2p= 0 .07$<br><br>21.50 (3.24 SD)<br>22.00 (4.03 SD)<br>non-sig,<br>$\eta 2p= 0 .00$<br><br>44.89 (8.25 SD)<br>44.00 (7.50 SD)<br>non-sig,<br>$\eta 2p= 0 .02$<br><br>48.38 (10.04 SD)<br>46.11 (9.32 SD)<br>non-sig,<br>$\eta 2p= 0 .03$	 <br>There were non-significant and small effects for between group and school x time across all four play types.	 <br>▲<br><br><br><br>▼<br><br><br><br>▼<br><br><br><br><br>▼	
Burgess & Ernst (2020), USA [27].<br><br>E: 84 children / 4 ECE<br><br>C: 24 children / 2 ECE	Controlled Before & After study	Play behaviours<br><br>The Penn Interactive Peer Play Scale consists of 32 items with 3 dimensions: play interaction, play disruption and play disconnection<br><br>Teachers and parents indicate frequency of behaviours on a 4-point Likert scale (never, seldom, often, always)	Adj means (SE)<br><br>Teacher:<br>Play interaction<br><br><br>Play disruption<br><br><br>Play disconnection	 <br><br>E: 23.44(0.31 SE)<br>C:17.75 (0.37 SE)<br><br><br>E:28.11 (0.67 SE)<br>C:25.19 (1.69 SE)<br><br><br>E:19.40 (0.53 SE)<br>C:15.88 (1.47 SE)	 <br><br>E:28.82 (0.32 SE)<br>C:26.13 (0.63 SE)<br>$p<.001$, $\eta 2= 0.12$<br><br>E:20.06 (0.48 SE)<br>C:25.22 (0.95 SE)<br>p <001, $\eta 2=0.19$<br><br>E:12.44 (0.32 SE)<br>C:15.17 (0.65 SE)<br>$p<.001$, $\eta 2= 0.12$	 <br><br>At post-test children in the nature ECE had significantly higher play interaction scores and lower play disruption and disconnection scores compare to the non-nature ECE.<br>(adjusted for pretest levels, age, gender, prior participation, and	 <br><br>▲<br><br><br><br>▲<br><br><br><br>▲	Weak

						part v. full-time participation)		
			Parent:<br>Play interaction<br><br>Play disruption<br><br><br>Play disconnection	E:25.77 (0.30 SE)<br>C:25.33 (0.75 SE)<br><br>E:29.82 (0.45 SE)<br>C:28.47 (1.20 SE)<br><br><br>E:17.75 (0.37 SE)<br>C:18.27 (1.27 SE)	E:27.15 (0.28 SE)<br>C:26.92 (0.58 SE)<br>p= 0.72, η2<.01<br>E:27.85 (0.45 SE)<br>C:28.45 (0.94 SE)<br>p= 0.57, η2< .01<br><br>E:16.06 (0.33 SE)<br>C:16.03 (0.69 SE)<br>p= 0.97, η2<.001	No significant differences between the nature and non-nature ECE at post-test.	▲<br><br>▲<br><br>▼	
Robertson et al (2020), Australia [41].<br><br>E: 15 children / 1 ECE<br><br>C: 15 children / 1 ECE	Controlled cross-sectional	Sociodramatic play<br><br>Smilansky Scale for the Evaluation of Dramatic and Socio Dramatic play (SSEDSP).<br><br>Observation of each child (6x5 minute intervals) and scored:<br>0=characteristic is not present<br>1=characteristic is present but to a limited degree<br>2=characteristic is present to a moderate degree<br>3=characteristic is present consistently and in many situations during the child's play	Sociodramatic play:<br><br><br><br>Role play<br><br><br><br><br><br>Make believe with objects<br><br><br><br><br>Actions and situations	E: 6.35 (1.96 SD)<br>C: 2.04 (2.65 SD)<br>t (28) = 5.07, p= 0.00)<br><br>E: 1.04<br>C: 0.34<br>SD= 0.16, p= 0.00, eta squared= 0.39<br><br>E: 0.92<br>C: 0.31<br>SD= 0.14, p= 0.00, eta squared= 0.42<br><br>E: 0.99<br>C: 0.34<br>SD=0.14, p= 0.00, eta squared= 0.44<br><br>E: 1.11	Mean diff= 0.86, (95% CI: - 2.04–6.35, eta squared = 0.47).	There was a significant difference between the sociodramatic play of children in nature ECE compared to the control The magnitude of the differences in the means was large. There were also significant differences in characteristic of Socio Dramatic Play.	▲<br><br>▲<br><br>▲<br><br>▲	Weak

		Total score was calculated using sum of each 5 min interval (score could be 0 - 18) and represented overall complexity of play	Persistence<br><br>Interaction<br><br>Verbal communication	C: 0.27 SD= 0.16, p= 0.00, eta squared= 0.50<br><br>E: 1.20 C: 0.34 SD= 0.14, p= 0.00, eta squared= 0.56<br><br>E: 1.20 C: 0.34 SD= 0.15, p= 0.00 eta squared= 0.53			▲<br><br>▲<br><br>▲	
**ECE natural playgrounds**								
Brussoni et al (2017), Canada [48].<br><br>E: 48 children / 2 childcare centres<br><br>Play: 16 children (sub-sample)	Uncontrolled before & after (mixed methods)	Play behaviours<br><br>Each child was observed twice over 30 min of outdoor play at baseline and follow-up by two researchers. Observations were coded as follows: prosocial behaviours (co-operative play, social conversation), antisocial behaviours (physical and verbal aggression, object possessiveness, rejected bids for engagement), lack of engagement in play (onlooking,	Play:<br><br>Prosocial behaviours<br><br>Antisocial behaviours<br><br>Lack of engagement in play<br><br>Channel surfing,<br><br>Child teacher interactions		OR: 2.81, (95% CI: 1.17-6.91), p< 0.05<br><br>OR: 1.40, (95% CI 0.47-4.13)<br><br>OR: 0.52, (95% CI: 0.24-1.14)<br><br>No change.<br><br>OR: 1.30, (95% CI: 0.65-2.57)	There were a significant intervention effects for play with natural materials and prosocial behaviour.<br><br>There were no significant intervention effects for the remaining play types.<br><br>Channel surfing and gender segregated play did not change.	▲<br><br>▲<br><br>▲<br><br>▶<br><br>▲	Moderate

		unoccupied), channel surfing (transitioning frequently between activities), child teacher interactions (teacher initiated, child-initiated, interruption by teacher), play with natural materials (natural loose materials, natural play elements), risky play (rough and tumble, height, mastery, unstable, speed, risk of getting lost), and gender-segregated play.	Play with natural materials<br><br>Risky play<br><br><br>Gender-segregated play<br><br>Solitary play		OR: 7.29, (95%CI: 1.53-38.09), p< 0.05<br><br>OR: 1.11, (95% CI: 0.55-2.27)<br><br>No change.<br><br><br>OR: 1.13, (95% CI 0.60-2.15).		▲<br><br><br>▲<br><br>►<br><br><br>▲	
Cloward Drown et al (2014), USA [36].<br><br>E: 24 children / 1 ECE (observed in 2 different playgrounds, natural vs manufactured)	Controlled cross-sectional	Dramatic Play<br><br>Smilansky Scale (modified) was used to code children's dramatic play. The scale uses 5 behaviors and persistence of a play episode to indicate dramatic play: imitative role-play, make-believe with objects, make-believe with actions and situations, interaction, verbal communication and persistence of play episode	Dramatic Play (%)<br><br><br>Playground type (natural vs manufactured)<br><br><br>Play props (natural, manufactured, none)	E: 12%<br>C: 10%<br><br><br>Pearson x2 = (3, 1006) = 12.19, p = 0.007)<br><br><br>Pearson x2 = (6, 802) = 23.09, p= 0.001)		Playground type and type of dramatic play were found to be significantly related with the natural playground affording more dramatic play than the manufactured playground.<br><br>A significant relationship was found between play prop use and dramatic play Natural play props were not used frequently or highly associated with dramatic play.	▲<br><br><br>▲<br><br><br>▲	Weak

		Social Play<br><br>MildredParten's (1932) stages of play were used to describe social interaction and maturity of play: unoccupied play, solitary play, onlooker play, parallel play, associative play, cooperative play.<br><br>Child's play was observed in 30-second intervals for ten-minute period. Observers recorded a child's location at the start of each 30-second interval and or the remainder of 30-second interval, the play types, persitance and location (natural, manufactured, none).	Social Play (%)<br><br>Playground type (natural vs manufactured)<br><br>Play props (natural, manufactured, none)	Pearson x2 = (3, 751), 5.07, p= 0.167<br><br>No association		There was no relationship between playground type and type of social play indicating both playgrounds provided similar affordances for social play.		
Luchs, & Fikus (2013), Germany [62].<br><br>E: 38 children / 1 ECE<br><br>C: 21 children / 1 ECE	Controlled cross-sectional	Play episodes and frequency<br><br>Observation - information on place, duration, social category of play and narrative was collected. The play episodes were then coded afterwards:	Number of play episodes<br><br>Duration of play episodes<br><br>0-5mins<br><br>6-10 mins	E: 3.05 ± 1.71<br>C: 5.57 ± 1.47.<br><br>E: 36%<br>C: 58%<br><br>E: 32%		During the 30 minutes observed, there were significantly different number of play episodes between the natural and contemporary playgrounds.		Weak

		**-play with**: functional play and constructional play.<br>**-play as**: well-known meaning and displays a different object within the child's play and imagination, orientation on role-models, not only copying but also developing their own play while realizing their own ideas, wishes and needs<br>**-play for**: play with rules, organizing activities of several players<br>**- others**<br>**- combination**		C: 35%				
			11-15mins	E: 12%<br>C: 7%				
			16-20mins:	E: 8%<br>C: 0%/				
			21-25mins	E: 5%<br>C: 0%				
			26-30mins	E: 8%<br>C: 0%				
			Frequency of play categories				▼	
			Play with	E: 1.45 ±1.37<br>C: 3.14 ±1.68<br>p= 0.000		Children in the contemporary playground engaged in significantly higher play episode categories. Combination was non-significant		
			Play as	E: 0.53 ±0.83<br>C: 0.62 ±0.97<br>p= 0.701			▼	
			Play for	E: 0.13 ±0.41<br>C: 0.52 ±0.68<br>p= 0.023			▼	
			Other	E: 0.24 ±0.49<br>C: 0.67 ±0.73<br>p= 0.022			▼	
			Combination	E: 0.71 ±0.8<br>C: 0.62 ±0.8<br>p= 0.677			▼	
			Combination Patterns of					

			play categories (%)<br>Play with<br><br><br><br>Play as<br><br><br><br>Play for<br><br><br><br>Other<br><br><br><br>Combination	 <br>E: 44.66 ±35.67<br>C: 56.18 ±27.45<br>p= 0.204<br><br>E:18.92 ±27.87<br>C: 11.78 ±23.28<br>p= 0.324<br><br>E: 3.23 ±10.46<br>C: 9.93 ±13.45<br>p= 0.056<br><br>E: 6.3 ±13.34<br>C: 11.45 ±12.31<br>p= 0.151<br><br>E: 26.9 ±32.71<br>C: 10.66 ±15.0<br>p= 0.012		Play for and combination play were significantly different. Combination play which was preferred by children in the nature playground.	▼<br><br>▼<br><br>▼<br><br>▼<br><br>▲	
Dyment et al (2013), Australia [42].<br><br>E: 120 children / 3 ECE<br>C: 40 children / 1 ECE	Cross-sectional	Play types<br><br>System for Observing Play and Leisure Activity in Youth (SOPLAY) was used to collect data on play types across various playground areas. The categories of play types were functional, constructive, symbolic, self-focused/looking on and talking.	Play types in natural areas<br><br>Functional (physical play activities)<br><br><br><br><br><br>Constructive (building play activities)	 <br>E:<br>ECE A= 24.0<br>ECE C= 58.3<br>ECE D= 52.2<br><br>C:<br>ECE B= N/A<br><br><br>E:<br>ECE A= 14.7<br>ECE C= 19.2<br>ECE D= 13.0<br><br>C:		Functional play was the most popular type of play in natural areas in the experimental schools. Symbolic play was infrequent and only observed in one experimental ECE.		Weak

			Symbolic (creative/ imaginative play)	ECE B= N/A<br><br>E:<br>ECE A= 8.0<br>ECE C= 0<br>ECE D= 0<br><br>C:<br>ECE B= N/A				
Morrissey et al (2017), Australia [43].<br><br>E: 28 children / 1 ECE<br><br>C: 28 children / same school as E.	Cross-sectional	Sociodramatic play episodes<br><br>Observation (2 independent researchers) using the Dramatic Play Data Collection Tool. The following play behaviours were coded:<br>- Play themes or roles were identified as present or absent in the episode: fantasy, domestic, occupational, conventional superhero or other.<br>- Frequencies of object substitutions<br>- Frequencies of imaginative transformations<br>- Frequencies of explicit metacommunications used to plan and organise play<br><br>Additional contextual	Fantasy<br><br>Domestic<br><br>Occupational<br><br>Superhero<br><br>Other<br><br>Relationship between sociodramatic play variables and context.<br>Object substitutions<br><br>Explicit metacommunication<br><br>Imaginative transformations	E: 10 / C: 4<br><br>E: 8 / C: 15<br><br>E: 1 / C: 3<br><br>E: 2 / C: 0<br><br>E: 0 / C: 2<br><br>$\chi2 = 21.71$, $p < 0.001$<br><br>$\chi2 = 10.04$, $p < 0.01$<br><br>$\chi2 = 6.63$, $p < 0.05$		There were significant associations between object substitutions, explicit metacommunication and imaginative transformations and the yard type (natural versus traditional).<br><br>Children from the natural playground engaged in longer episodes of	▲<br><br>▲<br><br>▲	Weak

		information was also collected				sociodramatic play episodes compared to children from the traditional playground and were more likely to engage in object substitutions, explicit metacommunication and imaginative transformations.		
**Natural elements within ECE**								
Zamani (2013), USA [37].<br><br>36 children / 1 ECE	Cross-sectional (mixed-methods – thesis)	Cognitive Play<br><br>Behaviour mapping - assesses individual cognitive play in the different zones. Children are observed for 7 days in 12 observation sessions during recess (11.30am and 4.15pm - lasted 45 minutes). The researcher scanned each zone and repeated for 4 rounds per recess. Childs location, gender, ethnicity, behaviour setting type, physical elements, cognitive play behaviour and teacher interactions were recorded. Each child was observed for 10 seconds and recorded for 20.	% time in play categories<br><br>Functional<br><br>Constructive<br><br>Exploratory<br><br>Dramatic<br><br>Games with rules<br><br>Functional<br><br>Constructive	**Natural:**<br><br>Within = 30.7; withinCog= 27.5<br><br>Within = 8.1; withinCog= 47.2<br><br>Within = 12.8; withinCog= 45<br><br>Within = 37.1; withinCog= 40.2<br><br>Within = 3.1; withinCog= 3.1<br><br>x= 281.70, 4***<br><br>**Mixed:**<br>Within = 35.2; withinCog= 35.2<br><br>Within = 4.5; withinCog= 29.1		All zones mainly afforded functional play opportunities. The natural zone afforded higher levels of dramatic, exploratory and constructive play compared to the other zones.	N/A	Weak

			Exploratory	Within = 10.9;<br>withinCog= 42.7				
			Dramatic	Within = 26.8;<br>withinCog= 32.5				
			Games with rules	Within = 13.9;<br>withinCog= 62.1<br><br>x= 201.46, 9***				
			Functional	**Manufactured:**<br>Within = 44.2;<br>withinCog= 37.3				
			Constructive	Within = 4.3;<br>withinCog= 23.6				
			Exploratory	Within = 3.7;<br>withinCog= 12.3				
			Dramatic	Within = 26.7;<br>withinCog= 27.3				
			Games with rules	Within = 6.8;<br>withinCog= 25.7<br><br>x= 224.86<br>3***				

Abbreviations: E= experimental; C= control; n= number; ECE = Early Childhood Education (includes preschools, day care, kindergarten etc.); SD= standard deviation; SE= standard error; CI= confidence intervals.

Effect direction explained:
▲: positive health impact
►: no change/ conflicting findings
▼: negative health impact
▲: positive health impact and statistical significance (p<0.05)
▼: negative health impact and statistical significance (p<0.05)

No arrow: no inferential statistics reported<br><br>Controlled before & after studies – difference between experimental and control group at follow-up (unless stated) or difference in change between experimental or control group. Uncontrolled before & after studies – change since baseline (unless stated). Controlled cross sectional – difference between experimental and control (unless stated). Cross-sectional – positive, negative or no association

Cognitive

Table S5. Nature-based ECE on cognitive outcomes								
**Study details** (Author, year and country)<br><br>**Sample size** (n of children / n ECE settings for exp and con)	**Study Design**	**Outcome and measurement**	**Units**	**Baseline or one time point (cross-sectional)**	**Follow-up (if applicable) or mean difference**	**Summary of Findings**	**Effect Direction**	**Quality Rating**
**Nature-based ECE**								
Agostini et al (2018), Italy [59].<br><br>E: 41 children / 7 teachers / 1 school	Controlled Before & After	Kuno Beller Developmental Tables completed by educators which assesses development in 8 developmental areas: Body Function, Awareness of the Surrounding	Language	T1 (Jan 2014)<br><br>E:11.01 (1.30 SD)<br><br>C:9.83 (1.53 SD)	T4 (May 2015)<br><br>12.88 (1.03 SD)<br><br>12.74 (1.24 SD)<br><br>p= 0.000; ηp^2= 0.42	There was a significant time x group interaction on children's language.<br><br>There were no significant differences between groups at T4.	▲	Weak

C: 52 children / 13 teachers / 1 school		Environment, Social and Emotional Development, Play, Language, Cognitive Development, Gross and Fine Motor Skills.	Cognitive development	E:10.94 (0.89 SD)<br><br>C:9.63 (1.35 SD)	12.49 (0.95 SD)<br><br>12.58 (1.31 SD)<br><br>p= 0.000; $\eta p2$= 0.51.	As above	▼	
Cooper (2018), United Kingdom (England) [56].<br><br>E: 13 children<br><br>C: 11 children<br><br>Children from the same school	Controlled before & after study	Communication<br><br>Assessed using FOCUS-34 (Focus on the Outcomes of Communication Under Six) which evaluates communication development. FOCUS - 34 is divided into 2 sections (34 items in total) and scored on a 7-point Likert scale.	Communicatio n<br><br>(median and range)	E: 206 (73)<br><br>C: 214 (93)	206 (73), Z=2.49 p=0.0013<br><br>214 (93), Z=2.85 p=0.004<br><br>U=54.5 p=0.694	No significant between-group differences at T2	▼	Weak
		The Devereux Early Childhood Assessment for Pre-schoolers, Second Edition (DECA-P2) consists of 38 items on a 5-point likert scale. The assessment measures protective factors and screen for behavioural concerns. The protective factors are divided into 3 subscales: initiative **self-regulation** and attachment/ relationships which form an overall measure of social and	Self-regulation (median and range)	E: 24 (22)<br><br>C: 23 (19)	25 (20); Z=1.48 p=0.138<br><br>24 (18); Z=1.63 p=0.102<br><br>**U=56.0 p=0.767**	No statistically significant between-group differences at T2 for self-regulation, initiative	▲	

		emotional wellbeing when combined.<br><br>Parent and teachers completed the form and they were asked to reflect on the child's behaviour for the previous 2 weeks.						
Cordiano et al (2019), USA [26].<br><br>E: 12 children / 1 ECE class.<br><br>C: 14 children / 1 class.<br><br>Children from the same school.	Controlled before & after study	Kindergarten readiness<br><br>Tool assessed letter number recognition, sorting and classifying information, counting, rhyming, and recognizing one's name. The skills were rated by the teachers as "Never," "Sometimes," "Often," or "Always".	Kindergarten readiness	T1 - baseline<br><br>E:19.09 (3.86 SD)<br><br>C:23.42 (3.44 SD)	T3 - endpoint<br><br>24.72 (2.87 SD)<br><br>26.79 (1.71 SD)<br><br>Within group: $\eta^2 p$= 0 .10 (small effect), p>0.05<br><br>Between group: F= 4.05, $\eta 2p$= 0.16, p> 0.05.	Non-significant and moderate effect for between group differences.	▼	Weak
Ernst & Burcak (2019), USA [30].<br><br>E: 34 children / 2 ECE<br>C: 43 children / 2 ECE	Controlled Before & After study	Curiosity<br><br>Curiosity Drawer Box task - There are a total of 12 possible points (1 point per drawer) for each of these three dependent measures (toys out, toys explored, toys engaged with further), with higher numerical scores indicating higher levels of the	Toys Taken Out:<br><br>Toys Explored:	E: 8.38 (3.39 SD)<br>C: 7.81 (4.19 SD)<br><br>E: 6.44 (3.09 SD)<br>C: 3.50 (2.71 SD)	Adj post-test (mean and SE)<br><br>9.61 (0.46 SE)<br>8.85 (0.40 SE)<br>p = 0.21,<br>ηp2 = 0.02<br><br>6.05 (0.66 SE)<br>6.24 (0.57 SE)<br>p = 0.83<br>ηp2 < 0.01	At post-test, there were no significant differences between the nature and non-nature groups for toys taken out or toys explored, toys engaged with was significant.<br><br>(controlled for pre-test, age, gender, and prior participation)	▲<br><br>▼<br><br>▲	Weak

		respective forms of curiosity. If a child returns to a drawer or toy after having already opened that drawer or interacted with that toy, they do not receive additional points.	Toys Engaged With:	E: 4.15 (2.60 SD)<br>C: 4.23 (2.89 SD)	7.61 (0.48 SE)<br>5.92 (0.42 SE)<br>p = 0.01<br>ηp2 = 0.09			
Burgess & Ernst (2020) [27].<br><br>E: 84 children / 4 ECE<br><br>C: 24 children / 2 ECE		Learning behaviours<br><br>Preschool learning behaviours scale which consists of 24 items with 3 dimensions: competence motivation; attention/ persistence and attitudes.<br><br>Teachers score on a 3-point Likert scale (doesn't apply, sometimes, apply, most often applies)	Adj means (SE)<br><br>Competence motivation<br><br>Attention/ persistence<br><br>Attitudes<br><br>Total	 <br>E:16.73 (0.45 SE)<br>C:19.53 (0.83 SE)<br><br>E:13.18 (0.37 SE)<br>C:+ SE)<br><br>E:11.11 (0.28 SE)<br>C:11.77 (0.39 SE)<br><br>E:36.53 (0.83 SE)<br>C:41.77 (1.51 SE)	 <br>E:20.41 (0.33 SE)<br>C:18.66 (0.65 SE)<br>p=0.02, n2=0.05<br><br>E:16.66 (0.30 SE)<br>C:16.13 (0.59 SE)<br>p=0.41, n2=0.01<br><br>E:12.74 (0.22 SE)<br>C:12.22 (0.42 SE)<br>p=0.27, n2=0.01<br><br>E:44.16 (0.68 SE)<br>C:41.76 (1.34 SE)<br>p=0.12, n2=0.02	At post-test, the nature ECE had significantly higher competence motivation compared to the non-nature ECE.<br><br>(adjusted for pre-test levels, age, gender, prior participation, and part v. full-time participation)	▲<br><br>▲<br><br>▲<br><br>▲	
Zamzow & Ernst (2020) [32].	Controlled Before & After study	Executive functions<br><br>Minnesota Executive Function Scale (MEFS) - conducted using an App, children perform a game like activity where they sort cards to boxes. This games changes commands to	Executive functions	E:41.78 (14.89 SD)<br>C:38.54 (14.40 SD)	Adj post-test (mean and SE)<br><br>50.86 (1.29 SE)<br><br>49.72 (1.73 SE)<br><br>p= 0.60, ηp2 < 0.01	No significant differences between the nature and non-nature groups when controlling for pre-test, age, gender, and prior participation.	▲	

E: 78 / 4 ECE<br>C: 44 children / 2 ECE<br><br>Wojciehowski & Ernst (2018) [31].		assess cognitive flexibility, inhibitory control, and working memory and provides an executive function total score.						
E: 75 children / 4 ECE	Uncontrolled Before & After study	Creative thinking<br><br>Thinking Creatively in Action and Movement (TCAM) consists of four activities that measure fluency, originality, and imagination.	Fluency<br><br>Originality<br><br>Imagination	E: 89.89 (17.76 SD)<br><br>E: 96.13 (20.16 SD)<br><br>E: 89.85 (17.68 SD)	104.76 (28.35 SD), p < 0.001<br><br>113.61 (36.58 SD), p< 0.001<br><br>99.99 (18.42 SD), p< 0.001	Significant improvements in fluency, originality, and imagination in the nature preschool from baseline to follow-up.	▲	
Ernst et al (2019), USA [29].<br><br>E: 78 children / 4 ECE		Resilience<br><br>Devereux Early Childhood Assessment for Preschoolers, Second Edition (DECAP2) - Parents and teachers evaluate 27 positive behaviors, which form 3 subscales: initiative, **self-regulation**, and attachment. Three subscales were converted to standard scores (T-scores) with a mean of 50 and SD of 10.	Teacher:<br><br>Self-regulation:	E:54.49 (6.00 SD)	56.78 (8.05 SD), p= 0.01	Significant improvements in self-regulation scores in the nature preschool from baseline to follow-up.	▲	
			Parent<br><br>Self-regulation:	E:49.31 (7.98 SD)	53.35 (9.34 SD), p= 0.01	Significant improvements in self-regulation in the nature preschool from baseline to follow-up.	▲	
Müller et al (2017), Canada [47].	Controlled before & after study	Executive functions<br><br>Working memory: the boxes task is a touch-	Working memory	E:25.38 (1.25 SE)<br>C:26.69 (1.18 SE)	E:20.85 (1.91 SE)<br>C:24.84 (1.87 SE)<br>p= 0.19, η2= 0.02)	At post-test there was a small and non-significant effect for working memory and	▲	Weak

E: 43 children / 1 nature-kindergarten<br><br>C: 45 children / 1 traditional kindergarten		screen operated, self-ordered search task designed to measure working memory.<br><br>Attention: Continuous Performance Test (CPT)- a computer based task that requires children to respond to stimuli by touching an animal on the touchscreen and to refrain from responding to a number of other stimuli types. The task lasted 5 minutes and included 200 stimulus of which 29 were targets. The number of correctly identified targets was used as performance indicator of directed attention.<br><br>Inhibition: The Head-Shoulders-Knees-Toes task (HSKT) - a task that involved children listening to commands and performing the opposite (e.g. touching head when researcher instructed them to touch their feet). Children were given a score out of 40.	Attention<br><br><br><br>Inhibition	E:22.67 (0.92 SE)<br>C:23.87 (0.86 SE)<br><br><br>E:28.96 (3.24 SE)<br>C:27.83 (3.16 SE)	23.70 (1.01 SE)<br>24.98 (0.94 SE)<br>p= 0.51, η2= 0.01<br><br>34.73 (2.34 SE)<br>33.44 (2.29 SE)<br>p= 0.76, η2= 0.00	attention. No effect for inhibition.	▼<br><br><br><br><br>▲	

		Social Skills Rating Scale (SSRS) completed by parents and teachers. This assesses the following social skills: cooperation, assertiveness, social responsibility and **self-control** and items assessing psychological health (internalising and externalising behaviour). Questionnaires were completed by teachers and parents. They were asked to indicate how often a behavior occurred (never, sometimes, very often).	Teacher<br><br>Self-control<br><br><br><br>Parent<br><br>Self-control	 <br>E:16.12 (0.56 SE)<br>C:14.71 (0.55 SE)<br><br><br><br>E:14.75 (0.54 SE)<br>C:14.68 (0.70 SE)	 <br>18.10 (0.56 SE)<br>13.52 (0.55 SE)<br>p= 0.00, η2= 0.32<br><br><br>15.78 (0.53 SE)<br>15.00 (0.69 SE)<br>p= 0.29, η2= 0.02	 <br>At post-test there was a large and significant effect.<br><br><br>At post-test there was a small and non-significant effect.	 <br>▲<br><br><br><br><br>▲	
Fyfe-Johnson et al (2019), USA [33].<br><br>E: 20 children / 1 ECE<br><br>C: 13 children (waitlist control or 2-hour nature-based, outdoor enrichment class provided by experimental ECE	Controlled cross-sectional	Child behaviour<br>SDQ: 25-items consisting of 5 domains: emotional problems, conduct problems, **hyperactivity/ inattention**, peer relationship problems, and prosocial behavior.<br>Parents rated their child on a scale of 0 to 2 per question (0=not true; 1=somewhat true; 2=certainly true). Overall score was	 <br>Hyperactivity/ inattention	 <br>E: 2.74 (2.27 SD),<br>C: 3.58 (2.27 SD)	Mean diff<br><br>-0.88 (95% CI: -2.71, 0.94)	 <br>Children in the nature ECE did not differ compared to the control.	 <br>▲	Weak

		calculated (sum of all domain scores except prosocial behavior; overall score range: 0-40). Prosocial was scored separately.						
Ernst (2014), USA [28].<br><br>E: 46<br>educators	Cross-sectional	Cognitive development<br><br>Questionnaire (not described) on importance of natural outdoor settings on children's cognitive, social, and physical development and their appreciation for the environment. Responses were provided on a five-point scale, ranging from one (strongly disagree) to five (strongly agree)	Cognitive development (1-5)	4.33 (1.30 SD), r= 0.05		There was no association between frequency of nature experiences and belief regarding importance of outdoor settings for cognitive development.	▲	Weak
**ECE natural playgrounds**								
Carrus (2012), Italy [60].<br><br>E: 16 children / 1 ECE	Cross-sectional	Visual spatial task (indicator of children's direct attention)<br><br>Children were asked to colour or to glue paper on to a drawing provided. Performances were evaluated by two independent coders.	Visual spatial task	No inferential stats provided.		Children exposed to free play in external green spaces exhibited a higher accuracy in the performance of the visual-spatial tasks compared to the control.	N/A	Weak
**Natural elements within ECE**								

Martensson et al (2009), Sweden [53].<br><br>E: 198 children / 11 ECE	Cross-sectional	Attention<br><br>The Early Childhood Attention Deficit Disorders Evaluation Scale (ECADDES, School) consists of 2 domains: inattention (32 items) and hyperactivity/ impulsivity (24 items) which are rated by two members of staff who observe the children in their daily routines. Each item is rated from 0-4 (0= child does not engage in the behavior at all, 1= behavior occurs one to several times per month, 2= behavior occurs one to several times per week, 3= behavior occurs one to several times per day, and 4= behavior occurs one to several times per hour) with a lower score indicating a lower occurrence. Rating are summed per child and raw scores converted into standard scores taking into account sex and age.	Hyperactivity/ impulsivity<br><br>Inattention	OPEC: Low Score= 1.59; High Score= 1.23, F= (-) 4.25, p= 0.069<br><br>OPEC: Low Score= 1.87; High Score= 1.46, F= (-) 7.38, p<.05		OPEC was significantly related to inattention dimension only:	▲<br><br>▲	Weak
**Garden-based intervention**								

Park et al (2016), South Korea [64].<br><br>E: 336 children /12 ECE<br><br>Science investigation abilities and attitudes= 68 children	Uncontrolled before & after	Scientific attitudes<br><br>The Scientific Attitude Survey revised by Lee (2000) was used. This consists of 27 questions on a five-point likert scale (strongly agree - strongly disagree) with 9 subcategories: curiosity, volunteerism and activeness, forthrightness, objectivity, openness, criticism, objectivity, cooperation, and patience. Teachers completed this questionnaire based on their daily observations. Higher scores indicate better scientific attitude.	Scientific attitudes (1-5)<br><br>Curiosity<br><br>Activeness<br><br>Forthrightness<br><br>Objectivity<br><br>Openness<br><br>Criticism<br><br>Judgement reservation<br><br>Cooperation<br><br>Patience	 <br>3.17 ± 0.98<br><br>3.13 ± 0.95<br><br>3.31 ± 0.77<br><br>3.07 ± 0.72<br><br>2.98 ± 0.64<br><br>2.79 ± 0.69<br><br>2.72 ± 0.74<br><br>3.13 ± 0.67<br><br>2.57 ± 0.77	 <br>4.11 ± 0.67, p=0.000<br><br>4.10 ± 0.65, p=0.000<br><br>4.07 ± 0.54, p=0.000<br><br>3.88 ± 0.69, p=0.000<br><br>3.55 ± 0.58, p=0.000<br><br>3.46 ± 0.59, p=0.000<br><br>3.42 ± 0.70, p=0.000<br><br>3.94 ± 0.65, p=0.000<br><br>3.77 ± 0.89, p=0.000	There were significant improvements in Science attitudes subcategories from baseline to follow-up.	▲	Weak
		Scientific investigations ability of younger children questionnaire revised by Lee (2000) was used. This consists of 21 questions on a five-	Scientific investigation abilities (1-5)<br><br>Prediction	3.11 ± 0.83	3.54 ± 0.63, p=0.002	As above.	▲	

		point likert scale (strongly agree - strongly disagree) with 5 subcategories: prediction, observation, classification, measurement, and discussion. A higher score indicates better investigation ability.	Observation<br><br>Classification<br><br>Measurement<br><br>Discussion	3.34 ± 0.92<br><br>3.25 ± 0.93<br><br>2.88 ± 0.97<br><br>3.04 ± 0.85	3.99 ± 0.67, p=0.000<br><br>3.93 ± 0.66, p=0.000<br><br>3.70 ± 0.68, p=0.000<br><br>3.55 ± 0.81, p=0.001			
Lillard (2016), USA [38].<br><br>E: 55 children / 1 ECE<br><br>Delay Gratification E: 34 children<br><br>Visual motor integration E: 39 children	Uncontrolled before & after	Delay Gratification<br><br>Participants were assessed individually. The researcher followed a script which involved the child receiving a treat if they waited for the researcher to complete a task. If they wanted the treat immediately, they could ring a bell for the researcher to come back but would get a smaller treat. Measurement was in seconds from when they rang the bell, or they reached 15 minutes.	Delay Gratification (seconds)	426.15	676.18, Non-sig	There was not a significant improvement from baseline to follow-up	▲	Weak

		Visual Motor Integration<br><br>Assessed using the Beery-Buktenica Developmental Test of Visual-Motor Integration 5th Edition (short form). This was a short pencil and paper test in which participants copy a sequence of shapes. Raw scores ranged from 0-20 and were transformed to standardized scores. Standard scores I have a mean of 100 (15 SD). Scores are age specific.	Visual Motor Integration (scores)	98.62	100.37, non-sig	As above		

Abbreviations: E= experimental; C= control; n= number; ECE = Early Childhood Education (includes preschools, day care, kindergarten etc.); SD= standard deviation; SE= standard error; CI= confidence intervals; OPEC= Outdoor Play Environment Categories.

Effect direction explained:
▲: positive health impact
►: no change/ conflicting findings
▼: negative health impact
▲: positive health impact and statistical significance ($p<0.05$)
▼: negative health impact and statistical significance ($p<0.05$)
No arrow: no inferential statistics reported

Controlled before & after studies – difference between experimental and control group at follow-up (unless stated) or difference in change between experimental or control group. Uncontrolled before & after studies – change since baseline (unless stated). Controlled cross sectional – difference between experimental and control (unless stated). Cross-sectional – positive, negative or no association

Nature connectedness

**Table S6. Nature-based ECE on nature connectedness**								
**Study details** (Author, year and country)<br><br>**Sample size** (n of children / n ECE settings for exp and con)	**Study Design**	**Outcome and measurement**	**Units**	**Baseline or one time point (cross-sectional)**	**Follow-up (if applicable) or mean difference**	**Summary of Findings**	**Effect Direction**	**Quality Rating**
**Nature-based ECE**								
Agostini et al (2018), Italy [59].<br><br>E: 41 children / 7 teachers / 1 school<br><br>C: 52 children / 13 teachers / 1 school	Controlled Before & After study	Kuno Beller Developmental Tables completed by educators which assesses development in 8 developmental areas: Body Function, Awareness of the Surrounding Environment, Social and Emotional Development, Play, Language, Cognitive Development, Gross and Fine Motor Skills.	Awareness of surrounding environment	T1 (Jan 2014)<br><br>E:11.35 (1.22 SD)<br>C:10.07 (1.80 SD)	T4 (May 2015)<br><br>13.20 (0.66 SD)<br>12.86 (1.09 SD)<br>$p= 0.004$,<br>$\eta p2= 0.30$.	There was a significant time x group interaction on children's awareness of surrounding environment.<br><br>There were no significant differences between groups at T4.	▲	Weak
Elliot et al (2014), Canada [46].<br><br>E: 21 children / 1 ECE<br><br>C: 22 children / 2 ECE	Controlled Before & After (mixed-methods)	Nature relatedness and environmentally responsible behavior<br><br>An activity where children played against the interviewer. 11 choices were presented (4 nature and 6 environmental behaviour) and the	Nature Relatedness (out of 8)<br><br>Environmentally responsible behavior (out of 12)	E: 6.43 (1.25 SD)<br>C: 6.05 (1.05 SD)<br><br>E:10.57 (0.93 SD)<br>C:10.59 (1.14 SD)	6.62 (0.97 SD)<br>5.82 (1.50 SD),<br>$p < 0.05$<br><br>10.71 (1.06 SD)<br>10.73 (0.83 SD),<br>$p< 0.40$	At post-test, there was a significant difference in nature relatedness scores between the groups.<br><br>At post-test, there was no significant between group differences.	▲<br><br>▼	Moderate

		child chose between 2 options.<br><br>Children received a score of 2 for choosing the more nature-oriented action or environmentally responsible option, and 1 for choosing the alternative option. The max score for nature relatedness was 8 and 12 for environmental behavior.						
Müller et al (2017), Canada [47].<br><br>E: 43 children / 1 nature-kindergarten<br><br>C: 45 children / 1 traditional kindergarten	Controlled before & after	As above.	Nature Relatedness (out of 8)<br><br>Environmentally responsible behavior (out of 12)	E: 6.37 (0.17 SE)<br>C: 5.82 (0.16 SE)<br><br>E:10.49 (0.18 SE)<br>C:10.29 (0.17 SE)	6.52 (0.18 SE)<br>6.14 (0.17 SE)<br>p= 0.22, η2= 0.02<br><br>10.49 (0.18 SE)<br>10.51 (0.17 SE)<br>p= 0.83, η2= 0.00	At post-test there was a small and non-significant effect<br><br>At post-test there was no significant effect	▲<br><br>▼	Weak
Nazaruk & Klim-Klimaszewska (2017), Poland [63].<br><br>E: 90 children (50 urban / 40 rural)	Uncontrolled before & after	Knowledge and skills of nature<br><br>Pre-test: A standard card test consisting of 6 illustrated worksheets with tasks for children to complete. Teachers explained and conducted the test.<br><br>Children's performance	Knowledge and skills of nature categorised into the following:<br><br>pre-test:<br>Low (0-9)<br>Average (10-14)<br>High (15-18)	City<br>Low= 12%<br>Average= 56%<br>High= 32%<br><br>Rural<br>Low= 0%<br>Average= 50%<br>High= 50%<br><br>p = 0.3	City<br>Low= 0%<br>Average= 28%<br>High= 72%<br><br>Rural<br>Low= 0%<br>Average= 20%<br>High= 80%<br><br>p = 0.8093	Children scored higher at post-test compared to pre-test.	▲	Weak

		was rated on a scale of 1 to 3 (1= nature skills have not been mastered, 3= nature skills have been fully mastered). Children could score a max of 18 points.<br><br>Post-test: Observation and a picture test consisting of 10 illustrated worksheet cards with tasks for children. A similar scoring to pre-test was used and the children could get a max of 30 points.	Post-test:<br>Low (0-15)<br>Average (16-23)<br>High (24-30)					
Yilmaz et al (2020), Turkey [65].<br><br>40 children / 1 ECE	Uncontrolled before & after	Biophilia<br><br>Adapted tool originally developed by Rice and Torquati (2013) below.	Biophilia Scores (out of 11)	19.78, 1.510 (SD), 0.239 (SE)	20.33, 1.309 (SD), 0.207 (SE)<br><br>Mean diff:<br><br>-0.55, 1.584 SD, 0.251 SE (95% CI: -1.057, -0.043), p= 0.034	There was a significant difference in the Biophilia scores from pre-test to post-test.	▲	Weak
Barrable et al (2020), UK (England, Scotland, Wales) [57].<br><br>E: 141 /12 ECE	Controlled cross-sectional	Connectedness to nature<br><br>The connectedness to Nature Index for Parents of Preschool Children (CNI-PPC) consists of 16-items and responses are given	Total CNI score<br><br>Enjoyment of nature	E: 4.22 (0.47 SD)<br>C: 3.92 (0.60 SD)<br><br>E: 4.41 (0.54 SD)<br>C: 4.05 (0.67 SD)<br>($\beta = 0.59$, $p = 2.61 \times 10^{-15}$)		Children attending nature nurseries scored higher for enjoyment and responsibility	▲<br><br>▲	Weak

C: 110 children / 6 ECE		on a five-item Likert scale ranging from "strongly disagree" to "strongly agree". It consists of 4 dimensions: enjoyment of nature, empathy for nature, responsibility toward nature and awareness of nature.	Empathy for nature<br><br>Responsibility toward nature<br><br>Awareness of nature	E: 3.78 (0.71 SD)<br>C: 3.63 (0.80 SD)<br><br>E: 3.96 (0.68 SD)<br>C: 3.85 (0.71 SD)<br>(β = 0.76, p = 2 × 10^{-16})<br><br>E: 4.45 (0.53 SD)<br>C: 3.98 (0.67 SD)			▲<br><br>▲<br><br>▲	
Giusti et al (2014), Sweden [54].<br><br>E: 11 children / 2 ECE<br><br>C: 16 children / 5 ECE	Controlled cross-sectional	Children's affinity with biosphere<br><br>The teacher presented children with image-based tasks (games) in which they had to select an image based on set questions. This assesses emotional and cognitive affinity to nature.	Emotional Affinity with the Biosphere<br><br>Cognitive Affinity with the Biosphere	E: 0.792 (0.121 SD)<br>C: 0.665 (0.154 SD),<br>p= 0.031, d= 0.916<br><br>E: 0.771 (0.134 SD)<br>C: 0.660 (0.133 SD),<br>p= 0.045, d= 0.845		Children with nature-rich routines score significantly higher than children with nature-deficit routines.<br><br>As above.	▲<br><br>▲	Weak
Rice & Torquati (2013), USA [34].<br><br>E: 68 children / 6 ECE<br><br>C: 46 children /4 ECE	Controlled cross-sectional	Biophilia<br><br>Interview consisting of 11-items which assess preference for being outdoors, enjoyment of sensorial aspects of nature, curiosity about nature, and interacting with nature.<br><br>Biophilic responses were scored 1 and non-	Biophilia Scores (out of 11)	E: 7.7 (2.3 SD)<br>C: 7.7 (2.4 SD),<br>p= 0.94		There was no significant difference between the nature and non-nature groups	►	Weak

		biophilic responses were scored 0.						
Ernst (2014), USA [28].<br><br>E: 46 educators	Cross-sectional	Development of environmental appreciation<br><br>Questionnaire (not described) on importance of natural outdoor settings on children's cognitive, social, and physical development and their appreciation for the environment. Responses were provided on a five-point scale, ranging from one (strongly disagree) to five (strongly agree)	Environmental appreciation (1-5)<br><br>Belief regarding difficulty in using natural outdoor settings<br><br>Belief regarding one's relationship with nature	4.43 (1.31 SD)<br><br>r= 0.83, p ≤ 0.05 b= 0.71, SE= 0.08, B= 0.83, p<.001<br><br>r= 0.31, p ≤ 0.05 b= 0.25, SE= 0.21, B= 0.11, p= 0.25		There was an association between frequency of nature experiences and belief regarding difficulty in using natural outdoor settings and belief regarding one's relationship with nature<br><br>Belief regarding difficulty in using natural outdoor settings was a significant predictor of use of natural outdoor settings with their preschool students, belief regarding one's relationship with nature was not.	▲<br><br>▲	Weak

Abbreviations: E= experimental; C= control; n= number; ECE = Early Childhood Education (includes preschools, day care, kindergarten etc.); SD= standard deviation; SE= standard error; CI= confidence intervals.

Effect direction explained:
▲: positive health impact
►: no change/ conflicting findings
▼: negative health impact
▲: positive health impact and statistical significance (p<0.05)
▼: negative health impact and statistical significance (p<0.05)
No arrow: no inferential statistics reported

Controlled before & after studies – difference between experimental and control group at follow-up (unless stated) or difference in change between experimental or control group. Uncontrolled before & after studies – change since baseline (unless stated). Controlled cross sectional – difference between experimental and control (unless stated). Cross-sectional – positive, negative or no association

Qualitative

Table S7. Findings from eligible qualitative studies

Theme	Subtheme	Studies	Quotes
Natural settings provide more affordances compared to traditional settings	Natural settings **enable** children to **diversify** their **play** (inc. imaginative, spontaneous, risky, manipulative, cognitive, exploratory and active play)	Dowdell et al (2011) [44]<br>Herrington & Studtmann (1998) [40]<br>Liu (2020) [39]<br>Puhakka et al (2019) [61]<br>Sandseter (2009) [52]<br>Wishart et al (2019) [45]<br>Zamani (2015) [37]	*"The children also invent themselves; when they have stimulus for their eyes, children invent it [activity] without your help. And it should be like this; some part should be like this. But you need to have stimulus. It's not enough to have a brown yard and a climbing frame. So, it [green yard] added somehow; they definitely had good games. They pretended that they had a campfire, they got the stones as sand pretended that they were on a trip. And their imagination was in use there, and when children use their brains, natural tiredness arises, and it did them good, a lot of good. Then rest comes naturally, and you have a good appetite and we're in the positive cycle. So they could use their imagination, and we encouraged them. We didn't prohibit them, we just advised them not to rip anything." (Puhakka et al, 2019)* [61].
	Natural settings **afford** children with **higher levels of risk** compared to traditional settings	Sandseter (2009) [52]<br>Streelasky (2019) [49]	*I like playing in the fallen logs and trees on the playground; it is so much fun, but a bit scary too! I like the big pile of sticks and logs that we made – it is for another fort that is going to be really high off the ground." (Streelasky, 2019)* [49].

	Natural settings **better support the use** and **improvements** in children's **imagination and creativity.**	Liu (2020) [39]<br>Streelasky (2019) [49]<br>Zamani (2015) [37]	*"I like being outside with my friends. We make shelters and we make up different games, like getting trapped on an island, or being on a boat and making our escape! I like doing science outside too – like different experiments, especially when the sun is out." (Streelasky, 2019)* [49].
	Natural settings **enable peers and teachers to have prosocial interactions**	Bjørgen (2016) [51]<br>Dowdell et al (2011) [44]<br>Liu (2020) [39]<br>Streelasky (2019) [49]	*"The children are shouting 'X… can't you catch us? Please catch us, try to catch us …'. The staffs join the situation and run after the children. The children are shouting 'Catch me … can't catch me' … There is excitement and the staff are running after the children, catching them and holding them before releasing them. The staffs have high energy, the children focus on the adults, avoiding being caught. The adults show empathy, holding and hugging the child when it is caught. The game is exciting and creates enthusiasm. A high level of physical activity is created, by climbing up, sliding down, running around and hiding in the tower to escape capture by the adults. They run at high speed and the children's body language shows that they are very much engaged in the game" (Bjørgen, 2016)* [51].
	Natural settings **increase child-initiated learning** compared to traditional settings	Dowdell et al (2011) [44]<br>Maynard et al (2013) [58]<br>Zamani (2015) [37]	Not available.
	Natural settings **enable children to** perceive themselves as **capable learners** compared to traditional settings	Dowdell et al (2011) [44]<br>Maynard et al (2013) [58]<br>Zamani (2015) [37]	*"[CogG] has poor concentration, sees herself as the baby, finds it difficult to sit and listen to story. She is extremely lacking in confidence … shy … she won't look at you indoors. With child-led learning she is totally engrossed and remains on task. Outside is the best learning environment for her … she remains on task. When outside she will come over and say 'I like this' and 'I like doing that', 'this is my favourite place'." (Maynard et al, 2013)* [58].
	Children have **increased contact with nature** enabling them to	Dowdell et al (2011) [44]<br>Liu (2020) [39]<br>Puhakka et al (2019) [61]	*"Especially about the forest floor mat, I remember that our children kept asking, 'what is it' and 'what's growing there', and explored it very carefully; they were almost lying on their stomachs there. Especially the*

	increase their knowledge of nature		*older ones, and they had a lot of questions about it." (Puhakka et al, 2019)* [61].
Natural and traditional settings provide similar affordances	**Opportunity for and frequency of risky play** is similar in both natural and traditional ECE settings.	Sandseter (2009) [52]	Not available.
Children's preferences of setting types	**Natural** environment is more diverse and engaging and **preferred** by children compared to traditional settings	Bjørgen (2016) [51]<br>Streelasky (2019) [49]	*"I like going outside and playing! I like playing with my friends, Sydney and Megan. We play hide and seek on the playground and hide in the forest in the logs and trees. I like outside because it's so fun and I really like to play. Sometimes I play with my sister too; I like all the colours outside and all the space." (Streelasky, 2019)* [49].
	**Mixed areas (**combining both natural with traditional elements) are **preferred** by children	Zamani (2015) [37]	Not available.
Restorative and invigorating effect of nature		Liu (2020) [39]<br>Puhakka et al (2019) [61]	*"Now it's become very difficult to finish playing. They would rather continue, and those who need to take a nap, they've had a nice, long time outdoors and nice games so they fall asleep more easily, and it affects their energy in the afternoon. Some children have very long days here. They come in the morning and stay until five o'clock; they seem to be somehow energetic and lively in the yard. This is new for us. The contrast to the previous yard is so great that the effects can be seen here very quickly." (Puhakka et al, 2019)* [61].

Additional file S6. Synthesis of qualitative and quantitative findings

**Themes from qualitative studies**	**Quantitative results**			
	**Nature-based ECE**	**ECE natural playgrounds**	**Natural elements within ECE**	**Garden-based interventions**
Natural settings enable children to **diversify their play**	Pretend play was higher.	Children engaged in more play with<br><br>natural elements, risky play, solitary play, dramatic play, sociodramatic play, functional and constructive play in natural playgrounds.	Compared to the mixed and traditional zones, the natural area afforded greater dramatic, exploratory and constructive play.	-
Natural settings **better support the use** and **improvements** in children's **imagination and creativity.**	All areas of creativity (fluency originality and imagination) improved in children who attended nature-based ECE.	Dramatic (inc sociodramatic) play was higher.<br><br>Functional and imaginative play was higher in traditional playgrounds.	-	-
Natural settings **enable peers and teachers to have prosocial interactions**	Social skills and social and emotional development were higher.<br><br>Unclear whether attachment was higher in children who attended nature-based ECE.	Social behaviour and social interactions were higher<br><br>More negative teacher and child interactions	-	There were improvements on emotional intelligence and prosocial behaviour.
Natural settings **increase child-initiated learning** compared to traditional settings	Attention, self-regulation, working memory, inhibition, total learning behaviours were better.	Children exposed to green space had higher visual spatial accuracy scores	Hyperactivity and inattention were better in setting with high quality nature versus low quality nature.	-

	Cognitive development was lower in children who attended nature-based ECE.	compared to children in the indoors setting.		
Children have **increased contact with nature** enabling them to increase their **knowledge of nature**	Nature relatedness, awareness of nature, knowledge of nature and awareness of the surrounding environment was higher.	-	-	-
	Environmentally responsible behaviour was lower.			
